# Instrument for Real-Time Digital Nucleic Acid Amplification on Custom Microfluidic Devices

**DOI:** 10.1371/journal.pone.0163060

**Published:** 2016-10-19

**Authors:** David A. Selck, Rustem F. Ismagilov

**Affiliations:** Division of Chemistry and Chemical Engineering, California Institute of Technology, 1200 E. California Blvd., Pasadena, CA, United States of America; Texas A&M University College Station, UNITED STATES

## Abstract

Nucleic acid amplification tests that are coupled with a digital readout enable the absolute quantification of single molecules, even at ultralow concentrations. Digital methods are robust, versatile and compatible with many amplification chemistries including isothermal amplification, making them particularly invaluable to assays that require sensitive detection, such as the quantification of viral load in occult infections or detection of sparse amounts of DNA from forensic samples. A number of microfluidic platforms are being developed for carrying out digital amplification. However, the mechanistic investigation and optimization of digital assays has been limited by the lack of real-time kinetic information about which factors affect the digital efficiency and analytical sensitivity of a reaction. Commercially available instruments that are capable of tracking digital reactions in real-time are restricted to only a small number of device types and sample-preparation strategies. Thus, most researchers who wish to develop, study, or optimize digital assays rely on the rate of the amplification reaction when performed in a bulk experiment, which is now recognized as an unreliable predictor of digital efficiency. To expand our ability to study how digital reactions proceed in real-time and enable us to optimize both the digital efficiency and analytical sensitivity of digital assays, we built a custom large-format digital real-time amplification instrument that can accommodate a wide variety of devices, amplification chemistries and sample-handling conditions. Herein, we validate this instrument, we provide detailed schematics that will enable others to build their own custom instruments, and we include a complete custom software suite to collect and analyze the data retrieved from the instrument. We believe assay optimizations enabled by this instrument will improve the current limits of nucleic acid detection and quantification, improving our fundamental understanding of single-molecule reactions and providing advancements in practical applications such as medical diagnostics, forensics and environmental sampling.

## Introduction

This paper describes a custom-built instrument and accompanying software for real-time digital nucleic acid amplification studies that can be used to develop, study, or optimize a wide variety of digital assays for numerous applications in a device-agnostic manner. Digital nucleic acid amplification works by partitioning a sample into many parallel individual samples. Of these partitioned samples, some may contain a target nucleic acid molecule (positive) while others do not (negative). By comparing the number of positive amplifications to the total number of partitioned samples, the absolute concentration of a sample can be calculated based on a Poisson distribution. Some of the advantages provided by a digital readout are that it can provide absolute quantification without standard calibration curves [1–3], it is robust to environmental conditions, including temperature, reagent quality and sample purity [4], and it can provide high resolution (< 1.5 fold change) [5], sensitivity [5], and accuracy [1] at low concentrations. Digital readouts enable precise counting of single molecules, including rare mutations and analyses of gene expression, which make them invaluable to assays that require detection at low concentrations, such as viral load in occult infections [3]. The digital method is also versatile; it can be coupled to many amplification technologies, including isothermal amplification chemistries, which in bulk reactions are limited in their ability to quantify very low concentrations of target molecules as a result of kinetic variations among samples [6].

Although reactions performed in a digital format provide these and many other advantages, few assay optimizations have been fully characterized. Thus, information on the quality of a reaction is confined to the end-point readout and little is known about how these reactions proceed in real time [2]. Currently, most optimizations for digital assays are performed in bulk by using the rate of the reaction as a proxy for efficiency [7,8]. However, rate doesn’t necessarily correlate with efficiency in all digital reactions and both “fate” and “rate” of individual digital amplification reactions should be measured in digital format [9]. Thus, to measure the digital efficiency of a reaction, optimization should be done in a digital format using real-time kinetic information for each compartmentalized reaction; to determine the performance of and variation between all independent single-molecule reactions.

One commercial option for performing real-time digital analyses on microfluidic devices is the Fluidigm Biomark HD. This instrument can be used to optimize digital assays because for each digital reaction, it can collect real-time traces (to determine the kinetic rate of the amplification) and melt curves (to determine false from true positive samples). While these real-time digital instruments have been used in a number of applications [1,2,10], they are limited to proprietary microfluidic devices with pre-established sample handling protocols. In addition to the commercial implementations, some laboratories have constructed real-time digital instrumentation for specific needs [11–17]. Many implementations of the digital format do not meet the requirements of commercially available real-time instruments; these include isothermal assays where the sample and amplification enzymes cannot be mixed until the sample has been partitioned [18,19] and single devices that include compartments of different volumes to increase the assay dynamic range [20].

An ideal real-time digital instrument would be fully customizable to work under a broad range of conditions, yet be simple enough for the average user to fully leverage its capabilities. Specifically:

- It should have accurate, precise and fully programmable temperature control to within 1°C across the full range of relevant temperatures.
- The imaging system should have sufficient resolution to accurately measure the kinetics of nanoliter amplification reaction volumes over a large field of view.
- It should incorporate multiple fluorescent channels to enable the analysis of multiplex reactions and non-standard chemistries.
- It should be compatible with collection and analysis software that is easy to use and capable of measuring the kinetics of reactions in any custom device.
- It should be easily adaptable to work with a wide variety of devices and architectures of varied sizes and materials.
- Its performance should be comparable with commercially available instruments that capture real-time performance in bulk reactions.
- It should be suitable for use with a wide variety of amplification chemistries.

**Fig 1:**
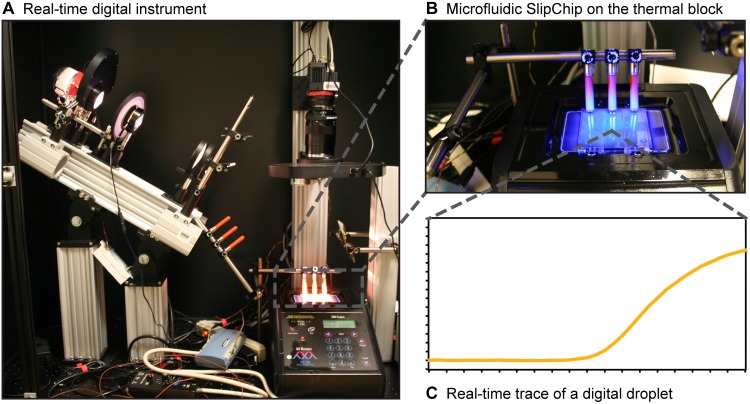
The large-format real-time instrument at different scales. (A) A photograph of the instrument in which its door has been removed for imaging; this door can be closed to block out light from the room. (B) A close-up view of the thermal block onto which the microfluidic devices are secured with a clamp to keep them in place. (C) A real-time trace of a digital reaction that was collected over the course of a real-time digital experiment from a single well on a microfluidic device.

To expand our ability to study how digital reactions proceed in real-time, including our capability to elucidate which factors affect the digital efficiency or analytical sensitivity of a reaction, we built a custom large-format digital real-time amplification instrument (Fig 1) that can accommodate a wide variety of devices, assays and conditions. This instrument has been used previously to optimize a loop-mediated isothermal amplification (LAMP) assay for hepatitis C virus (HCV) quantification [9] and to develop a method to both quantify and genotype HCV infections in a single step [21]. In this paper, we provide the schematics of the instrument used in these previous studies and we validate its performance using well characterized chemistries (real-time digital reverse transcription PCR) and a previously validated microfluidic device (SlipChip). We compare instrument performance to an Illumina Eco real-time PCR system using HCV RNA as the template. We also provide the relevant calibration and performance characteristics of the instrument, and the complete custom software suite used to collect and analyze data retrieved from the instrument.

## Results and Discussion

### Temperature control design and calibration

To obtain reliable real-time data from nucleic acid amplifications in either a bulk or digital format, temperature control must be precise and accurate. We have shown previously that while digital reactions can be robust to wide temperature changes [4] the kinetics of a reaction will vary as a function of temperature. Others have constructed thermal control units using Peltier elements [12–14], however, to increase reliability and accuracy we chose instead to modify an existing thermocycler. We used a PTC-200 thermocycler with a stated temperature range of -5°C to 105°C and accuracy of ± 0.3°C. This specific model was chosen because it can be fully controlled by third-party software through an ASCII control interface. Although it is no longer manufactured, it is widely available on the used-equipment market, and customization is simplified because the heated block is physically separated from the base instrument.

The thermocycler in its standard state is incompatible with custom devices because they are standardized to the well-plate format; thus, we customized the thermocycler block. We designed and machined from aluminum an *in situ* thermal block with the same thermal mass as a standard 96-well block to retain the rated ramp rate of the PTC-200 thermocycler (which is up to 3°C/s). The thermal block assembly contains a set of four different Peltier elements: a heatsink, a circuit board, thermal transfer sheets, and thermistors. The thermal block is kept in electrical isolation and thermal contact through the use of a Tgard K52 polyimide sheet in conjunction with a Tgon 805 graphite sheet, and the temperature of the custom block is reported to the thermocycler with three different 10K ohm thermistors. We assembled the block this way to closely match the standard thermal assembly to maintain optimal performance and require only minimal calibration.

**Fig 2:**
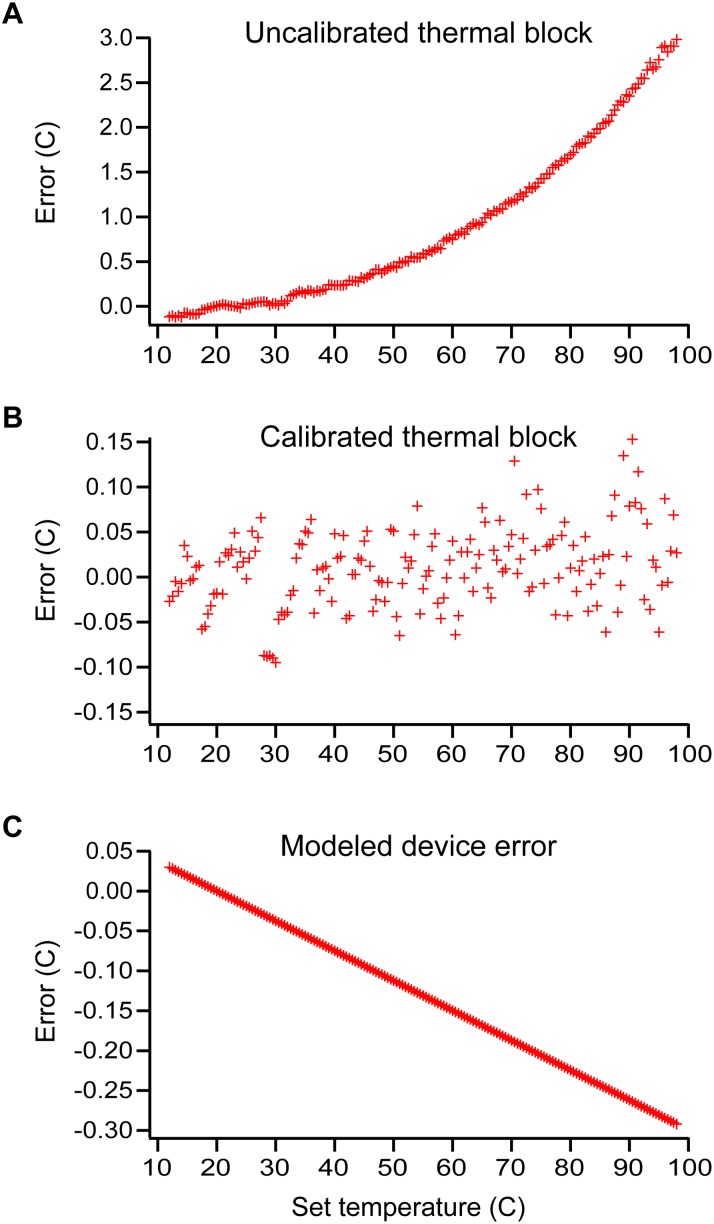
Temperature characterizations of the modified thermal block used in the PTC-200 thermocycler (A–B) and a Comsol-model to determine the on-device error (C). The deviation in the actual and reported temperatures are provided for the uncalibrated thermal block (A) and for the block after calibration (B). The Comsol-modeled deviation (C) provides the difference between the actual block temperatures and the theoretical reaction temperatures of the well volumes in a SlipChip microfluidic device. Error values are the difference between the thermocycler-reported thermal block temperature and either the actual thermal block temperatures (measured by a type K thermocouple) or the modeled device temperature; a positive number indicates the thermal block temperature is higher than the device temperature.

The temperature calibration experiments were performed over the range of 12–98°C with a temperature interval of 0.5°C. As expected, at room temperature the reported and actual readings were in close agreement because this class of thermistors has a defined resistance of 10K ohms at 25°C. As the temperature deviates from 25°C, the difference between the actual and reported temperatures of the block can vary by as many as 3°C (at a set temperature of 98°C) (Fig 1A). This is caused by a slight mismatch in the performance curves of the chosen thermistor as compared with the thermistor for which the thermocycler was originally calibrated. To compensate for this mismatch, a sixth-order polynomial correction is performed in the custom GUI based software, which brings the actual and reported differences of the custom thermal block to within 0.15°C (Fig 2B). This correction makes the difference in actual and reported temperatures lower than the rated 0.3°C variation of the thermocycler and thus falls well within desired performance characteristics; thus validating both the accuracy and precision of our custom *in situ* thermal block.

Calibration of the block does not control for the temperature of a reaction done on a device in the instrument. The temperature of a device may differ from the block as a result of convective cooling because a device is heated only from one side. In our lab, we primarily use custom SlipChip devices, and no commercially available temperature-sensing elements could be easily adapted; however, the sizes and geometries of SlipChip devices are well known and highly accurate, so the temperature error for SlipChip devices can be modeled using Comsol multiphysics (Fig 2C). The model was a stationary experiment with the same resolution and range as the calibration experiments we performed with the block. The Comsol model was based on a 1” x 3” x 0.02” soda-lime glass SlipChip with a thermal conductivity of 1.38 W/(m*k), as defined by the Comsol materials library, that was separated from the thermal block with a 50 μm gap filled with mineral oil having a thermal conductivity of 0.162 W/(m*k) [22] and a convective cooling rate of 10 W/m^2^K, the derivation of which is described in S4 File. The convective cooling rate can vary based on a wide variety of factors; using these parameters, the temperature difference between the block and the SlipChip device deviates -0.29°C from the set temperature at maximum block temperature (98°C). This offset is well within the required performance characteristics of our instrument.

### Optical design and validation

**Fig 3:**
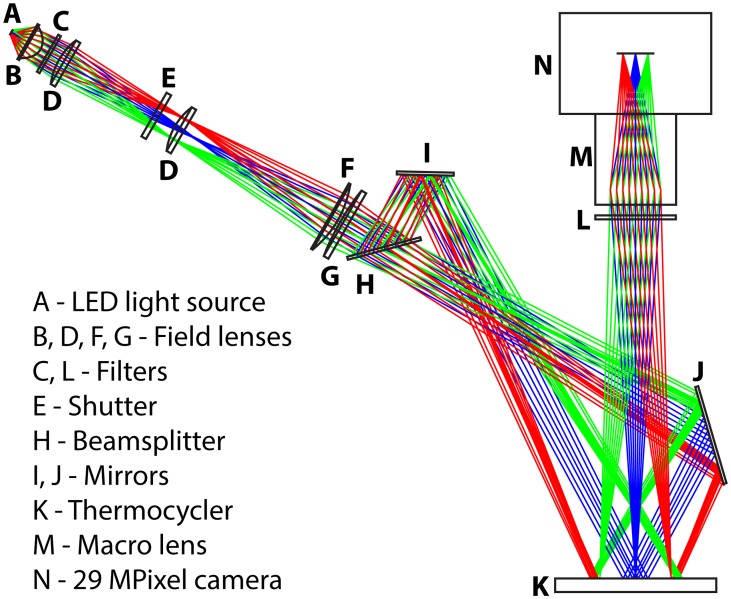
A schematic showing a scale representation of the physical layout of the custom real-time digital nucleic acid amplification instrument. Colored lines indicate ray paths.

The optical system of the instrument was designed to provide high illumination intensity over the full field of view (~5500 mm^2^) (Fig 3). We chose to prioritize lighting intensity over lighting uniformity because uniformity deviations can be corrected after imaging with simple flat-field corrections. We chose a white LED spotlight module because it has a dense power delivery (rated 1175 lumens from a total area of 64 mm^2^), which allows us to use common and reasonably sized optics. Although there are light sources that can provide greater illumination intensities, these lights would have a larger surface area and thus would require the field optics that direct the light to be much larger to compensate, which reduces the number of compatible standard lenses necessitating custom optics and much larger excitation fluorescence filters. In this custom real-time instrument, the largest cost in the optics is the fluorescence filters. Because filter cost is proportional to filter area, it is advantageous to keep the filters as small as possible.

The standard illumination geometry has 0-degree angle of incidence (perpendicular to the surface of device) because this minimizes gradients in the illumination field. Because the bottom of the device in this system is inaccessible due to the presence of a thermocycler, to use an angle of incidence of 0 degrees, we would need to image and illuminate from the same lens. Although this is commonly done, it creates a strong specular reflection of the excitation beam, which requires better emission filter blocking, and there are no commercial lenses of this type that can image the full field of view at high numerical aperture. Thus, to allow us to use standard optics, we set the angle of incidence at 27.5 degrees. To eliminate illumination gradients caused by the non-zero angle of incidence we added a beam splitter and mirror system (Fig 3). This geometry provides added flexibility; if a single light source provides insufficient illumination intensity, a second optical source and optics can be set up in lieu of a beam splitter and mirrors for a maximum of four separate light sources possible. In our system, we found that a single source provided sufficient illumination intensities with power values of up to 1 mW/cm^2^ of power with the 475 nm channel, 4.3 mW/cm^2^ with the 560 nm channel, and 4.6 mW/cm^2^ with the 630 nm channel. At the time this instrument was constructed, there was only one color (white 3000K) available for the LED module, however, there are currently many additional colors available. Different colors may better suit certain applications by shifting the higher power to the 475 nm channel.

**Fig 4:**
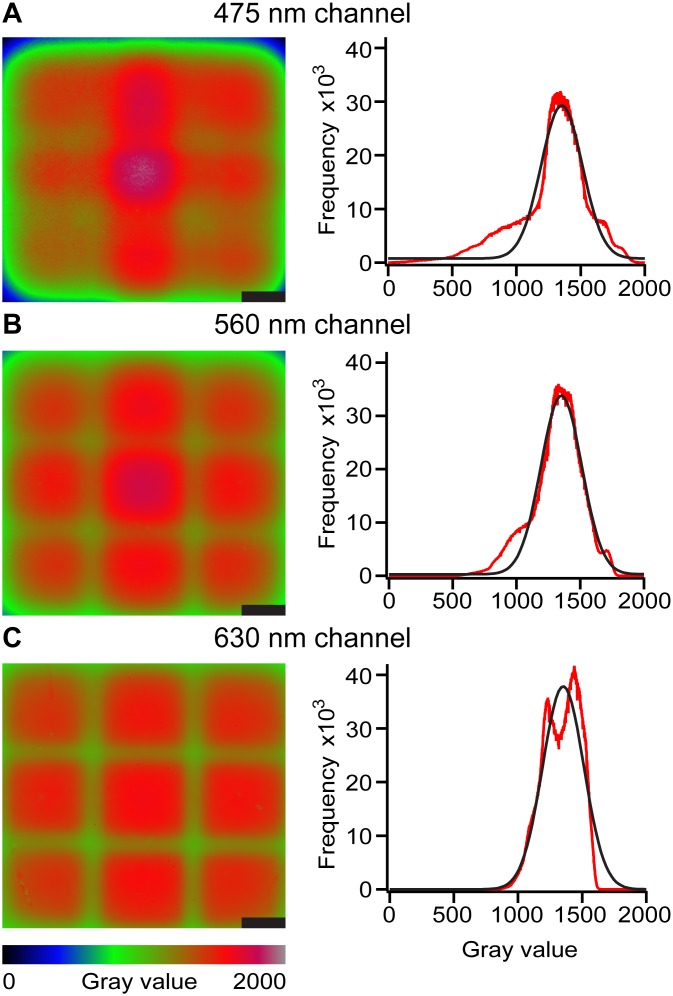
Results of the optical characterization of the real-time digital nucleic acid amplification instrument. Heat maps (left) and histograms (right) of custom fluorescence standards show the uniformity of illumination over the field of view as quantified in each of three fluorescent channels. Results from each channel were scaled to equalize each channel’s average gray value. The gray values from the heat maps and histograms are truncated to between 0 and 2000 as reported by the instrument’s built-in camera at each channel: (A) 475 nm, (B) 560 nm, and (C) 630 nm. Scale bars are 10 mm.

To validate the illumination uniformity at different excitation wavelengths, full-field fluorescence standards were created and imaged. Results from each channel were scaled by a constant to equalize each channel’s average gray value. In this system, we used non-apochromatic optics, so the focal ranges for each of the excitation wavelengths vary and illumination is not uniform over each of the channels. The 475 nm channel is both the most used and the lowest power, so it was aligned to have the highest power density while still maintaining sufficient uniformity; this was achieved by focusing the channel down to slightly less than full field, which reduces the illuminated area. This is not a problem for imaging as long as the reactions being imaged do not extend past the illuminated area. With this alignment, all channels achieve sufficient uniformity (Fig 4). The best-fit Gaussian curves for each channel have full width at half max values of ~228 units, or 17% of the average Gaussian value. A flat-field correction easily compensates for this variation while only minimally impacting noise.

### Software design and implementation

The software we created for the instrument was written to be amenable to a wide variety of different amplifications, analyses, and device geometries. Temperature programs are established by mixing ramp, temperature, and cycle steps in any order or combination, and imaging parameters are fully adjustable to any combination of exposure times and fluorescent channels in one of three imaging modes: (i) cycle-based imaging as done in standard quantitative PCR, (ii) time-based imaging where images are taken at set intervals throughout an experiment, or (iii) combination imaging where images are taken at set intervals only during a certain temperature step. The timing of the images can be set in one of two ways. If either time-based or combination imaging is used, the timing is controlled by software and is based on the time since the start of the previous acquisition. Thus, if an imaging step is longer than an imaging interval, image acquisition is continuous throughout the experiment. If imaging is cycle-based, the timing of image acquisition is controlled by the thermocycler. The thermocycler is set on a program; when it reaches an imaging step, it is allowed to proceed until there are 2 seconds remaining, at which time the running program is paused, the temperature is held, all defined images are acquired, and then the program resumes. If the imaging step occurs during a step which is cycled, an image is acquired during each cycle. Melt temperature data can be automatically collected after any experiment by defining a low temperature, high temperature, resolution, and relevant imaging parameters. As a quality assurance mechanism, we implemented an error-checking system into the software; if a required variable is either left undefined or improperly configured, the software will inform the user of the required changes before a run can be started.

The software enables full control over all of the instrument components to tune the instrument settings to a specific application. The camera control and configuration are set up to be agnostic to equipment models. Any GigE-compliant camera can be used with the software and all functions that are defined by the GenICam specification are controllable. A simplified configuration is provided in the software for defining exposure time, camera gain, framerate, and region of interest. The thermocycler can be configured to maintain a set temperature for a period prior to the start of any program. This can accommodate devices that need to be maintained in a specific temperature range prior to the start of an experiment, such as isothermal reactions that are active at room temperature [18]. Control over the positions and interactions of the filter wheels allows the user to define and name any combination of emission and excitation filters for imaging.

**Fig 5:**
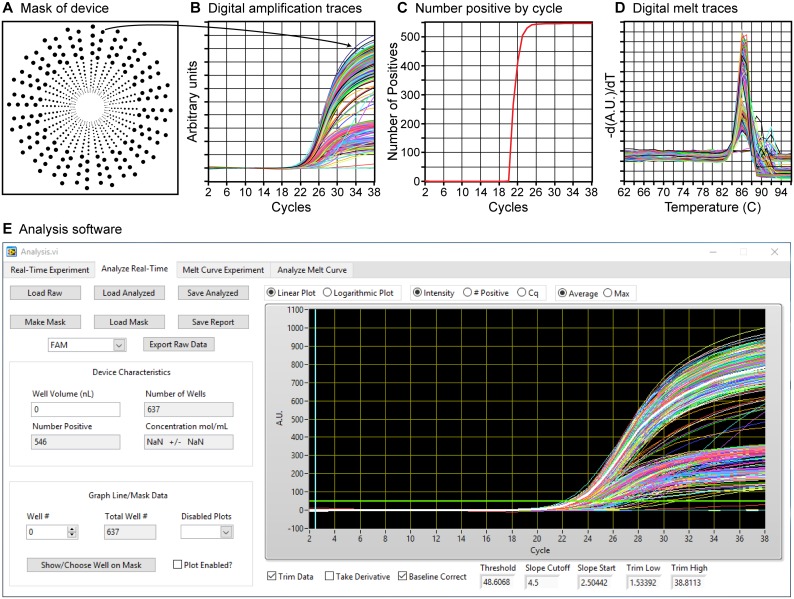
Types of data output provided by the custom analysis software of the real-time digital nucleic acid amplification instrument after a multivolume PCR reaction using lambda DNA on a multivolume SlipChip device with a 15 s exposure time. Each graph (B–D) was exported as line art and scaled. (A) An image depicting the mask created to define the locations of each compartmentalized reaction on a multivolume microfluidic device. (B) Baseline-corrected amplification traces from each of the reaction wells on the microfluidic device. Two intensity groups result because in this multivolume microfluidic device there are two well depths (the two larger volumes are 100 μm deep and the two smaller volumes are 50 μm deep) [20]. The arrow shows the correlation of a single compartmentalized reaction (A) to its real-time trace (B). (C) A graph depicting the number of positive reactions as a function of amplification cycle from the data generated in (B). (D) A graph depicting the negative derivative of the collected melt curve traces from each of the positive reactions. (E) A screenshot of the analysis software analyzing the real-time data shown in panel (B).

The software also contains a full suite of tools for data analysis. Results from an analysis of digital multivolume real-time PCR experiments [20] using lambda DNA as a template are shown in Fig 5 with each graph (Fig 5B–5D) directly exported from the analysis software as line art and scaled. These results were obtained by first creating a mask of the sections of the device containing reactions of interest (Fig 5A). A mask-creation tool is used to define the locations of compartments in images collected during an experimental run by using several built-in functions, including: thresholding, edge detection, region selection, removal of features based on area, automated removal of features on the edge of the image, and the ability to both “paint in” and “paint out” features. To account for potential device shifting during the experiment, the software has built-in tracking that recreates the mask with each individual step of the amplification and correlates relocated spots among the images based on nearest-neighbor calculations. During an analysis, each compartmentalized reaction is tracked across all of the stages of amplification and a full suite of statistical data is collected at each step for each region defined by the mask. The average value of each compartmentalized reaction is then plotted as a function of assay progress producing real-time curves for each individual reaction with full correlation back to the mask region as shown in Fig 5B. Additional software functions enable baseline correction, thresholding for determination of the quantification cycle (Cq), plotting the number of positive wells as a function of assay progress (Fig 5C), plotting Cq frequency as a function of assay progress, the determination of the derivative of any dataset and the generation and export of reports and datasets.

Melt curve data is initially processed in the same manner as amplification data, via creation of a mask and individually analyzing its corresponding regions (Fig 5D). The data can then be processed using functions such as: data smoothing via algorithms, determination of the negative derivative of the dataset, configurable peak melt temperature detection, correlation of melt curves to amplification curves including only showing melt curves from positive reactions, report generation, and ability to export any generated plot or dataset.

### Digital real-time PCR validation

We validated the overall performance of the instrument in a real-time digital reverse transcription PCR experiment using HCV RNA as the template. We used a previously validated microfluidic SlipChip device [9] and well-characterized chemistry [9,20,23] to isolate the performance of the instrument and compare it to an Illumina Eco real-time PCR system. The assay was run at three concentrations (each separated by a 100x difference) in parallel on microfluidic SlipChip devices. In the lowest concentration tested, the wells of the device were stochastically loaded with HCV RNA molecules; in the higher concentrations, all wells contained many copies of the target. The lowest concentration was calculated at 6 x 10^4^ ± 8 x 10^3^ molecules of HCV RNA per milliliter of reaction solution with a total of 202 positive reactions out of a possible 1218 after 40 cycles of amplification. This concentration corresponds to 0.18 copies of HCV RNA loaded per 3 nL digital compartment. The two concentrations that were 100x and 10,000x higher therefore correspond to 18, and 1800 copies of HCV RNA loaded per 3 nL digital compartment. Melt curve analysis was performed on each of the 202 positive reactions; all but five had a melt temperature indicative of the correctly amplified product. The error in the assay associated with false positive reactions accounted for 1 x 10^3^ copies/mL, which is much smaller than the 8 x 10^3^ error estimated by Poisson statistics, thus signifying suitable assay performance.

**Fig 6:**
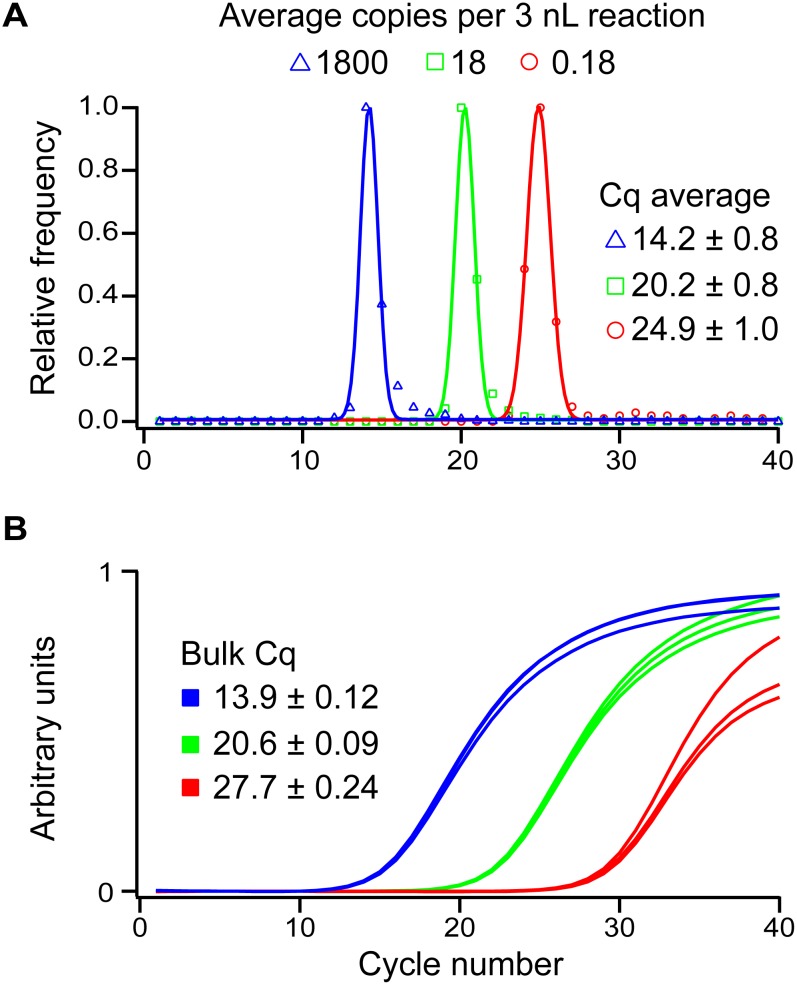
Comparison of digital (A) and bulk (B) results of real-time reverse transcription PCR experiments of HCV RNA at three different concentrations. Digital and bulk experiments were run at the same three concentrations (each separated by a 100x difference). The exposure time used in the experiment was 20 s. In (A), symbols show a histogram of the relative frequency of Cq values obtained in the experiment for the denoted concentration; solid lines depict the Gaussian fit for each concentration. Points denote the calculated histogram of Cq values at each concentration. Average Cq values are the maximum of the Gaussian fit and the errors are the full width at half maximum value of the Gaussian fit. (B) Bulk traces of reverse transcription PCR of HCV RNA. Average Cq values are shown; error denotes S.D. among the replicates (N = 3).

Because we have a known change in concentration between the three tested concentrations, we can run a variety of checks to ensure that the assay performs as expected kinetically. The first analysis that we ran was to look for the variation among replicates at the same concentration. Each of the three different bulk concentrations were run in triplicate, and the standard deviation of the replicates was less than 1% of the Cq value (Fig 6B) in each of those reactions, indicating that the kinetic rate within each concentration was highly reproducible and the assay was suitable for quantitative analysis. In addition to analyzing the kinetic reproducibility of replicates, the real-time instrument can also be used analyze reproducibility by measuring the Cq difference at different concentrations. Under ideal amplification conditions, concentration should double after each cycle. Assuming perfect amplification, the expected Cq difference between concentrations can be calculated based on the equation log_2_*x* where *x* is the fold change in concentration between the two samples. For an amplification reaction performed in a bulk format using a 100-fold change we expect a Cq difference of 6.6. The results of the experimental validation closely match this estimate. The Cq difference between the high and middle concentrations was 6.7 and the Cq difference between the middle and low concentrations was 7.1 (Fig 6B).

When the same comparative analyses are run on the real-time digital results, we observed similar effects. Because the experiments were run on a microfluidic device, hundreds of reactions were run at each concentration with the observed Cq values closely modeling a Gaussian distribution. The standard deviation of the Gaussian fit of each concentration in digital varies between 4% and 6%. This variation is higher than the 1% deviation observed in the bulk reactions, however, each concentration can be uniquely identified and is well separated.

The results using digital also compared favorably to the results performed in bulk on a commercial instrument. Because the assay was run with the digital device wells fully loaded with template molecules at the high and middle concentrations, the Cq values in digital should match those in bulk at these concentrations. Indeed, when comparing the digital averages to the bulk averages, we observed a Cq difference of 0.3 at the high concentrations, and a Cq difference of 0.4 at the middle concentrations. At the low concentration, we expect to see a deviation in Cq values. Because the low concentration is run in the digital regime on-device, each compartment that contained an HCV RNA molecule had a concentration of 1 copy/3 nL. In bulk, using the same concentration of RNA, the concentration of templates in solution was 0.18 copies/3 nL. Therefore, we would expect the on-device concentration to be 5.6 times higher, which would result in a predicted Cq difference of 2.5. When the bulk and digital Cq values at the low concentration are compared, the Cq difference is 2.8; a deviation (0.3) from the expected value that is within the same range as what we observed in the high and middle concentrations.

When we compare Cq differences using digital reactions at different concentrations, we expect Cq difference of 6.6 between the high and middle concentrations and a Cq difference of 4.2 between the middle and low concentrations. Indeed, when we ran these experiments we observed differences of 6.0 and 4.7. From these results it is clear that the digital real-time instrument is able to provide informative and reproducible results about the kinetics of amplification on digital microfluidic devices.

### Digital real-time isothermal validation

Many amplification chemistries are compatible with digital assays and could benefit from knowledge of kinetic information and digital optimization. Loop-mediated isothermal amplification (LAMP) has been used extensively in a digital format, however, the amplification efficiency of many LAMP reactions is consistently less than expected [4,9,19,24]. Because reaction efficiency is consistent, quantification can still be performed in the absence of optimization, however optimizing LAMP reactions performed in a digital format would both increase confidence in the final calculated concentrations and lower the current limits in detection and quantification. Such optimization is not possible using bulk reactions because changes in reaction rate do not necessarily correlate with changes in reaction efficiency [9]. We have shown previously [9] using the real-time digital instrument described herein, that the efficiency of a reverse transcription LAMP reaction targeting HCV RNA can be optimized quickly and effectively by using real-time information combined with digital information. The amplification efficiency of HCV RNA through the usage of real-time digital data was improved from ~20% to ~70%, significantly increasing the confidence of the calculated concentration and lowering the detection limit of the assay [9].

**Fig 7:**
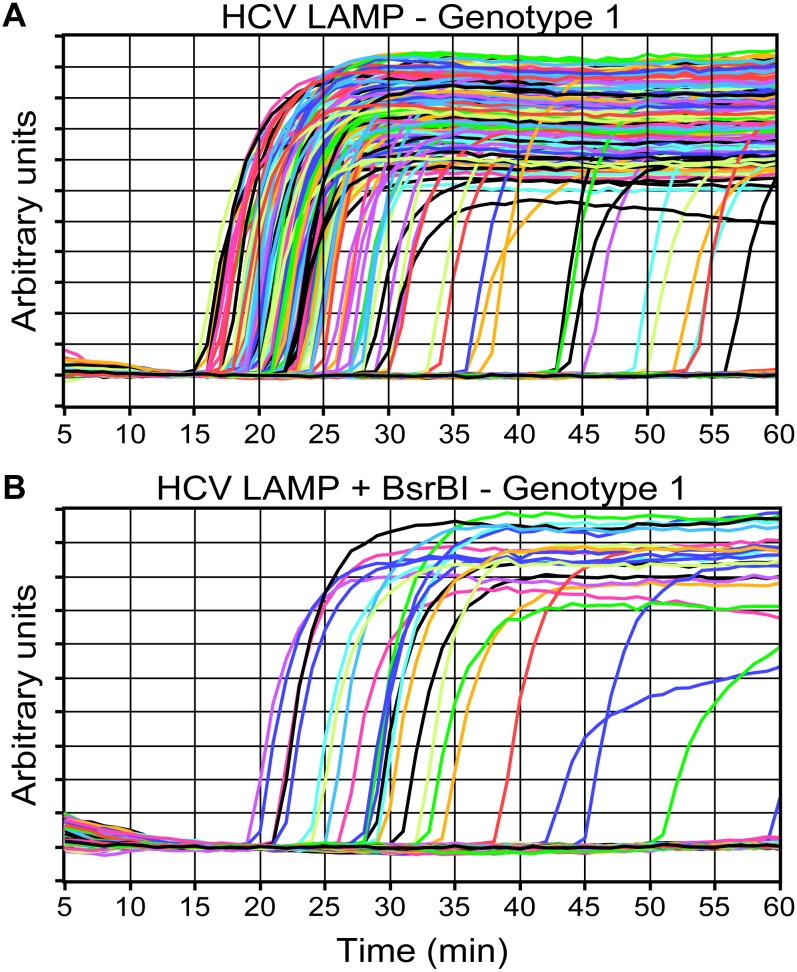
Results of reverse transcription LAMP of HCV RNA reactions in the absence (A) and presence (B) of a competing restriction digestion enzyme, BsrBI. In (A) the reaction is allowed to proceed normally with high reaction efficiency and fast reaction rates. In (B) the reaction efficiency and rate are significantly lower because BsrBI degrades the template.

We have also previously used this instrument to optimize and verify an assay to simultaneously quantify and genotype HCV RNA [21]. This was done by performing a competition reaction between LAMP amplification and genotype-selective degradation of the template using restriction enzymes. In bulk assays, the restriction enzymes were shown to delay the reaction, however, in the digital format the competition reaction significantly decreased the number of positive digital reactions. Using the real-time digital instrument, we were able to show that the digital competition reactions on HCV genotype 1 (Fig 7B) that amplified did so with a decreased reaction rate compared to a positive control (Fig 7A). We further showed that the decrease in reaction rate in digital format was similar to the decrease in reaction rate that was observed in bulk reactions. Having this detailed information about the kinetics of individual reaction volumes enabled us to establish that the so-called “fate” of a reaction (i.e. whether or not the reaction will proceed) is determined in the first steps of a reaction; and once that fate is determined, the competition reaction affects only the rate. This paper and our paper showing the lack of correlation between reaction speed and analytical sensitivity[9] wouldn’t have been possible without this instrument. We are continuing to use this instrument in our lab to develop and enable new analytical capabilities for a wide range of sample and device types.

## Conclusions

We developed an instrument for real-time digital nucleic acid amplification studies and accompanying software that can be used to develop, study, or optimize a wide variety of digital assays for numerous applications, including medical diagnostics, forensics and environmental sampling. The instrument is device-agnostic and it is compatible with a variety of nucleic acid amplification chemistries, including PCR, reverse transcription PCR, LAMP, and reverse transcription LAMP in a competition reaction with restriction enzymes. We validated this real-time instrument using two types of microfluidic devices, one that contained a large number of reactions in compartments of the same size, and a second device that processes larger volumes with a range of different reaction sizes. We also presented a simple, intuitive, GUI-based software package that allows the user to easily set up, collect, and analyze digital experiments using real-time fluorescence information from a wide variety of possible devices with a total precision-heated and viewable area of ~5500 mm^2^.

The capabilities provided by this instrument will be invaluable for researchers who wish to track the progress of reactions at high resolution with a large field of view and its use will enable the study and optimization of a wider variety of digital reactions in real time. Although the error in the temperature control is well within our required performance, this and other device error can be corrected via in-software calibrations that can be customized for any specific device. The instrument was designed to be modular so that it is amenable to further customization, such as to increase illumination intensity, enable heating of non-flat devices, increase the field of view, or increase the resolution of the captured area as new technologies are introduced they can be easily incorporated without a significant engineering effort. We hope that by sharing the details of this instrument and software, others will be able to construct their own purpose-built custom instruments or modify existing commercial solutions to characterize and optimize digital reactions, making digital methods even more powerful.

## Materials and Methods

### Chemicals and materials

All chemicals were purchased from commercial sources. The LoopAmp^®^ RNA amplification kit (Eiken Chemical Co., Ltd., Japan) was purchased from SA Scientific (San Antonio, TX, USA). The LoopAmp^®^ RNA amplification kit contains 2X Reaction Mix (RM) (40 mM Tris-HCl pH 8.8, 20 mM KCl, 16 mM MgSO_4_, 20 mM (NH4)2SO_4_, 0.2% Tween20, 1.6 M Betaine and dNTPs 2.8 mM each), Enzyme Mix (EM) (mixture of Bst DNA polymerase and AMV reverse transcriptase), and distilled water (DW). Bovine serum albumin (BSA) was purchased from Roche Diagnostics (Indianapolis, IN, USA). Bio-Rad (Hercules, CA, USA) SsoFast Evagreen Supermix, Phage lambda DNA (500 μg), SUPERase In RNase Inhibitor (20 U/μL), mineral oil (DNase, RNase, and Protease free), and tetradecane were purchased from Thermo Fisher Scientific (Hanover Park, IL, USA). All primers were produced by Integrated DNA Technologies (Coralville, IA, USA). Dichlorodimethylsilane was purchased from Sigma-Aldrich (St. Louis, MO, USA). Photomasks were designed in AutoCAD 2013 and ordered from CAD/Art Services, Inc. (Bandon, OR, USA). Soda-lime glass plates coated with layers of chromium and photoresist were ordered from the Nanofilm (Westlake Village, CA, USA). The PTC-200 thermocycler was purchased second-hand off of Ebay (San Jose, CA, USA) and was manufactured by MJ Research (Waltham, MA, USA). The Tgon 805, Tgard K52, and PR103J2 thermistors were purchased from Digikey (Thief River Falls, MN, USA). The illumination optics were purchased from Edmund Optics (Barrington, NJ, USA) (part numbers 46–685, 48–247, 48–372, 63–496, 48–904, 48–451, 48–453). The LED light source, power supply, and heat sink were purchased from Future Electronics (Montreal, Canada). The filter wheels were purchased from Finger Lakes Instrumentation (Lima, NY, USA). The camera for the instrument was purchased from Vision Systems Technology (Vista, CA, USA). The camera lens was purchased from Digitalrev.com (Kowloon, Hong Kong). The green fluorescent filter set was purchased from Semrock (Rochester, NY, USA). The other filter sets for Texas red and Cy5 dyes were purchased from Omega Optical (Brattleboro, VT, USA). The thermocouples and digital acquisition device were purchased from Omega Engineering (Stamford, CT, USA). The various aluminum extrusions, filter holders, lens holders, optical posts, and other ancillary equipment to construct the shell of the instrument were purchased from either Thorlabs (Newton, NJ, USA), Edmund Optics (Barrington NJ, USA), Newport Corp. (Irvine, CA, USA), Grainger (Lake Forest, IL, USA), or 80/20 (Columbia City, IN, USA). Custom CNC milled parts were ordered from Protolabs (Maple Plain, MN, USA). A detailed list of parts and their sources is provided in S2 File.

### Microfluidic device design

A multivolume rotational SlipChip device was used as a model to illustrate some of the capabilities of the instrument for performing amplification reactions on custom microfluidic devices. Use of this particular multivolume device was first published in Shen *et al*. [20]. Briefly, the device has four different sized wells of 1, 5, 25, and 125 nL. There are 160 different wells of each volume on the device; the two smaller wells have a depth of 50 μm and the two larger wells have a depth of 100 μm.

A single-volume microfluidic device was used to perform reverse-transcription PCR (RT-PCR) and compare the performance of the instrument in different concentration regimes with traditional quantitative PCR. This particular single-volume device is a lightly modified version of the device used by Sun *et al*. [19]. It contains 1280 reaction wells each with a 3 nL volume. All wells and channels in the device are etched to a depth of 50 μm.

### Microfluidic device fabrication

Microfluidic devices were fabricated using standard photolithography followed by wet chemical etching with hydrofluoric acid [25]. After etching the devices to the proper depths and drilling access holes with a diamond-coated bit, devices were subjected to a previously described silanization process [26].

### Assembling and loading microfluidic devices

The microfluidic devices were assembled under degassed oil consisting of a combination off 75% mineral oil and 25% tetradecane (v/v). Both the top and bottom sections of the microfluidic device were immersed in oil before being aligned under a stereoscope (Leica, Wetzlar, Germany). The devices were then clamped together using standard 1” binder clips. Solutions were introduced into the microfluidic devices by drawing the solution to be loaded on the device into a pipettor (Eppendorf, Hamburg, Germany), placing the end of the pipette tip into the pre-drilled access holes on the device to create a seal, and applying a pressure of 0.1 atm.

### Illumination setup

The illumination setup of the instrument was designed to be composed of standard and easily obtainable illumination and optical components. A white LED module (LSX8-PW30, Fig 3A) was used as the optical source of the instrument and provides 1175 lumens of flux from an 8mm x 8mm area. Five lenses were set up to direct the light to the imaging platform; these consisted of: a 40 mm aspheric lens (46–685, Fig 3B), two 50 x 125 mm plano-convex lenses (48–247, Fig 3D), a 75x 200 mm plano-convex lens (48–372, Fig 3F), and a 75x 500 mm plano-convex lens (63–496, Fig 3G). The excitation wavelengths are chosen by a five-position filter wheel (CFW-1-5, Fig 3C) that holds a selection of three 50.8 mm excitation filters: a 475 nm centered filter (FF02-475/50-50.8-D), a 560 nm centered filter (560QM55), and a 630 nm centered filter (630QM50). After passing through the field lenses, the light is split by a 50–50 beamsplitter (48–904, Fig 3H) before either reflecting off of a 75 X 75 mm mirror (48451, Fig 3I) or a 75 x 100 mm mirror (48–453, Fig 3J). The optics are designed to provide demagnified and slightly defocused light to an imaging area of ~72 x 72 mm. The light must be slightly defocused to attain a relatively constant illumination intensity over the full area as the source is a 3 x 3 grid of smaller light emitting diodes.

### Imaging setup

The instrument uses a six-position filter wheel (CFW-6-6, Fig 3L) that is equipped with a set of three 79 mm emission filters: a 540 nm centered filter (FF01-540/50-79-D), a 645 nm centered filter (645QM75), and a 695 nm centered filter (695QM55). With these filter sets, we are capable of imaging dyes such as fluorescein, Texas red, and Cy5. We used a VX-29MG-M2-A0-F-2 29-megapixel camera (Vieworks, Anyang, South Korea) that utilizes a KAI-29050 6576 x 4384 monochrome sensor (ON Semiconductor, Phoenix, AZ, USA). This camera has a 23 mm sensor with a pixel pitch of 5.5 x 5.5 μm, a 12-bit low noise amplifier, a GigE vision interface, and a standard Nikon F lens mount. The lens used is a Makro-Planar T* 100mm f/2 ZF.2lens (Ziess, Oberkochen, Germany) which has a wide f/2 aperture and the ability to focus on an area as small as 48 x 72 mm. The camera is triggered via an OMB-DAQ2408-2AO data acquisition board (Measurement Computing, Norton, MA, USA) through an analog output with millisecond accuracy. The data acquisition board is also used to control the shutter of the system and collect temperature data. This imaging setup enables us to image the full 72 x 72 mm field of view wherein each pixel is equivalent to 16 x 16 μm real-world resolution yielding a spatial resolution of 4500 x 4500 pixels (20 MP). Complete schematics of the real-time digital instrument are available in S1 File.

### Fluorescence standards

Because the total viewable area on the custom-built instrument is rather large, no commercially available fluorescence standards were found that were large enough to encompass the full field of view in a single image. Fluorescence standards for the 475 nm and 560 nm emission channels were created by coating one side of a 72 mm x 76.2 mm piece of soda-lime glass with Rust-Oleum 1932830 or 1959830 paint. A fluorescent standard for the 630 nm channel was created by gluing a Rosco Roscolux #2001 storaro red filter between two pieces of soda-lime glass cut to a size of 72 mm x 76.2 mm with Loctite^®^ #349 optical adhesive.

### Temperature control

Temperature control is provided by a PTC-200 thermocycler (Bio-Rad, Hercules, CA, USA). The thermocycler was chosen for its simple ASCII programming interface, which is via an RS-232 port, and because blocks are easy to interchange. The heating block used in the instrument is a heavily modified 96-well alpha block. This custom thermal block was designed and fabricated from aluminum and contains a raised flat section measuring 72 x 76.2 mm that has the same thermal mass as the block included in the system. The block is in thermal contact with the Peltier elements of the alpha block using first a ceramic filled polyimide sheet (Tgard K52) and second a thermally conductive graphite sheet (Tgon 805). The block incorporates three 10K ohm at 25°C thermistors (PR103J2) in the standard alpha block locations, which were soldered directly to the circuit board. All temperature measurements are recorded with type-K thermocouples (5TC-TT-K-40-36).

### Temperature readout of thermal block

Temperature readings of the thermal block are collected with a 5TC-TT-K-40-36 thermocouple attached to an OMB-DAQ2408-2AO digital acquisition instrument. The thermocouple is in thermal contact with the heated block using Arctic MX-4 thermal paste. Temperature readings for calibration and validation of the thermal block can be collected between a range of 4°C and 98°C with a resolution of 0.1°C and a frequency of 1 kHz. In the validation experiments performed here, data was between 12°C and 98°C at a resolution of 0.5°C collected by setting the thermocycler to hold the desired temperature, and waiting until the temperature stabilized within a 0.1°C for a total of 30 s. The temperature readings collected over the 30 s period were then averaged and used for all calculations.

### Nucleic acid amplification reagents

PCR experiments were carried out using 2x SsoFast EvaGreen supermix with a 1 μM concentration of primers and varied concentrations of template. The template used in the multivolume PCR experiments was lambda DNA with primer sequences (GAA TGC CCG TTC TGC GAG, TTC AGT TCC TGT GCG TCG). The temperature profile used in those experiments was a 5-minute melt at 95C followed by 40 cycles of 1 min at 95°C, 30 s at 58°C, and 30 s at 72°C. The PCR experiments done to compare reactions in digital to reactions in bulk used hepatitis C virus (HCV) RNA with primer sequences (GAG TAG TGT TGG GTC GCG AA, GTG CAC GGT CTA CGA GAC CTC). The temperature profile used in those experiments was a 30-min 50°C reverse transcription step followed by a 3-min melt at 94°C followed by 40 cycles of 1 min at 94°C, 30 s at 62°C, and 30 s at 72°C.

### Collection software

Simple and easy-to-use GUI based custom software was designed for the real-time digital instrument using the LabVIEW 2013 development suite with the vision acquisition, OpenG, and System Controls 2.0 add-on packages. Control over the camera is achieved using the National Instruments IMAQdx driver to control any GIGE compliant camera. Control over the filter wheels is achieved using the FLI Software installation kit (4/14/2010 update) and version 1.104 of the FLI SDK. Control over the thermocycler is achieved using the ASCII commands outlined in the PTC-200 user manual and the RS-232 driver in LabView. The data acquisition board is controlled through Omega DAQ software (v. 6.22) utilizing the ULx LabView drivers. The software saves imaging data in the PNG format, temperature data as tab delimited text files, and experimental parameters in binary files. The software includes a variety of different modules for the collection of amplification data, melt temperature data, temperature calibration data, as well as acting as a generalized large-format fluorescence imager. The software also allows the user to save experimental programs and settings, including full experimental profiles, camera settings, and temperature calibration settings for individual assays.

### Analysis software

Simple and easy-to-use GUI based custom software was designed for the analysis of collected data using the LabVIEW 2013 development suite with the vision acquisition, OpenG, System Controls 2.0, and the report generation toolkit for Microsoft Office add-on packages. The software is able to open and analyze all generated data from the collection software including the ability to recall all temperature data, experimental conditions, and presets. The software includes a variety of different modules for analysis of the data including all amplification and melt temperature data, mask generation for defining digital reaction locations in custom microfluidic devices, and report generations and export to Microsoft Word and Excel. The analysis software is also able to export raw or analyzed data as well as any plot in a variety of formats.

The software is able to create independent amplification curves from each discreet digital reaction through the usage of a user created mask which defines the reaction location in space. Once a location is defined, full statistical information about that independent reaction is available for that point during the reaction. A full suite of tools is available for creating the mask including thresholding, edge detection, size discrimination, location selection and rejection, and manual editing. If during the course of the amplification some reaction volumes move outside of their masked area, tracking software can be activated in the software which rebuilds the mask at each point in the amplification based on the process defined by the user and correlates the reaction volumes between images based on nearest neighbor calculations. After amplification curves have been established, baseline correction, Cq calling, report generation, and correlation of each reaction trace to its discreet reaction volume can be performed. Melt curve analysis can be performed in much the same way, although, added features such as peak calling, correlation to amplification reactions, and curve smoothing can be performed. Source code for the software described herein is located at dx.doi.org/10.6070/H4N29V0S. A software users’ guide is in S5 File.

## Supporting Information

S1 File Schematics of instrument. Engineering drawings, optical simulation results, and alignment procedures for the real-time digital instrument.
(PDF)

S2 File Detailed parts list. A detailed list of all components used in construction of the real-time digital instrument.
(PDF)

S3 File Raw Data. The raw data used to make all of the figures in the manuscript.
(XLSX)

S4 File Derivation of convective cooling rate. A derivation of the convective cooling rate used in the device temperature simulations.
(PDF)

S5 File Software users’ guide. A brief guide detailing how to use the collection and analysis software.
(PDF)
