# Supplementary material for: Instrument for Real-Time Digital Nucleic Acid Amplification on Custom Microfluidic Devices
Source: PLoS One. 2016 Oct 19;11(10):e0163060. doi: 10.1371/journal.pone.0163060 (PMC5070811; doi:10.1371/journal.pone.0163060)
Supplement: S1 File — Engineering drawings, optical simulation results, and alignment procedures for the real-time digital instrument. (PDF) [file pone.0163060.s001.pdf]

S1. Schematics of instrument. Engineering drawings, optical simulation results, and alignment procedures for the real-time digital instrument.

Instrument for Real-Time Digital Nucleic Acid Amplification on Custom Microfluidic Devices

David A. Selck, Rustem F. Ismagilov
Optical Design by David H Tracy

Approach

- SLM-TB Radiance checked in Non-Sequential Zemax model
- Actual design done in Sequential mode for efficiency
  - Use approximation of SLM-TB emission
  - Actual uniformity will be slightly lower, corners slightly rounded
- Illum optics axis tilted 30° to allow use of standard 45° Beamsplitter.
- Low cost, off-the-shelf optics employed, no chromatic correction.
- Magnification chosen to generate 65mm wide uniform zone
  - Mag can be reduced to increase irradiance on smaller subzone
  - Dual Counter-propagating Beams for uniformity
  - B/S could be removed to use single beam

Emission Optics

- Camera and lens shown for completeness, not to scale
- No details of Emission Optics are covered in this document.
  - Will depend on results of Objective Lens testing.

Philips Fortimo SLM Gen2 Tight Beam Module

- Contains LED array similar to Luxeon S (8mm dia dome)

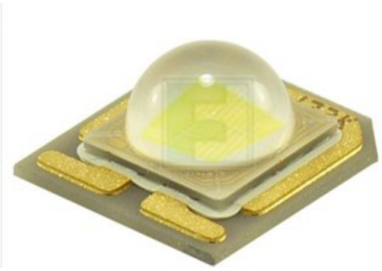

Note: Effective source plane is 3.0 mm above heat sink mounting base of SLM and centered on module.

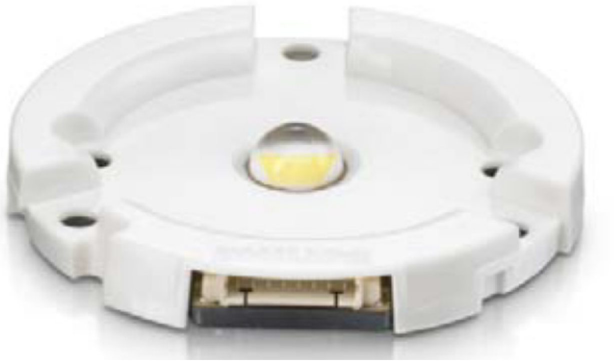

- 3x3 close packed die covering
  ~ 4.8 x 4.8 mm effective area
- Up to 1300 lumens total flux
  - ~ 4 watts total across spectrum
  - ~ 150 to 300mW per excitation band *deliverable to target*
- Excellent optical source modeling support provided by Philips
- As usual, LEDs show large temperature dependence
  - Keep source on constantly to stabilize

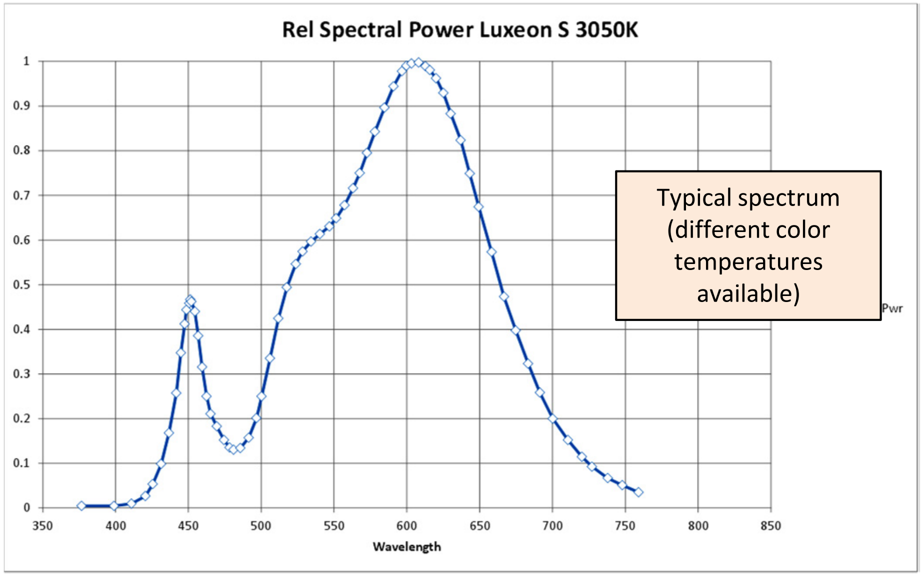

Illum System v2.1
[Filter Wheels Not Shown]

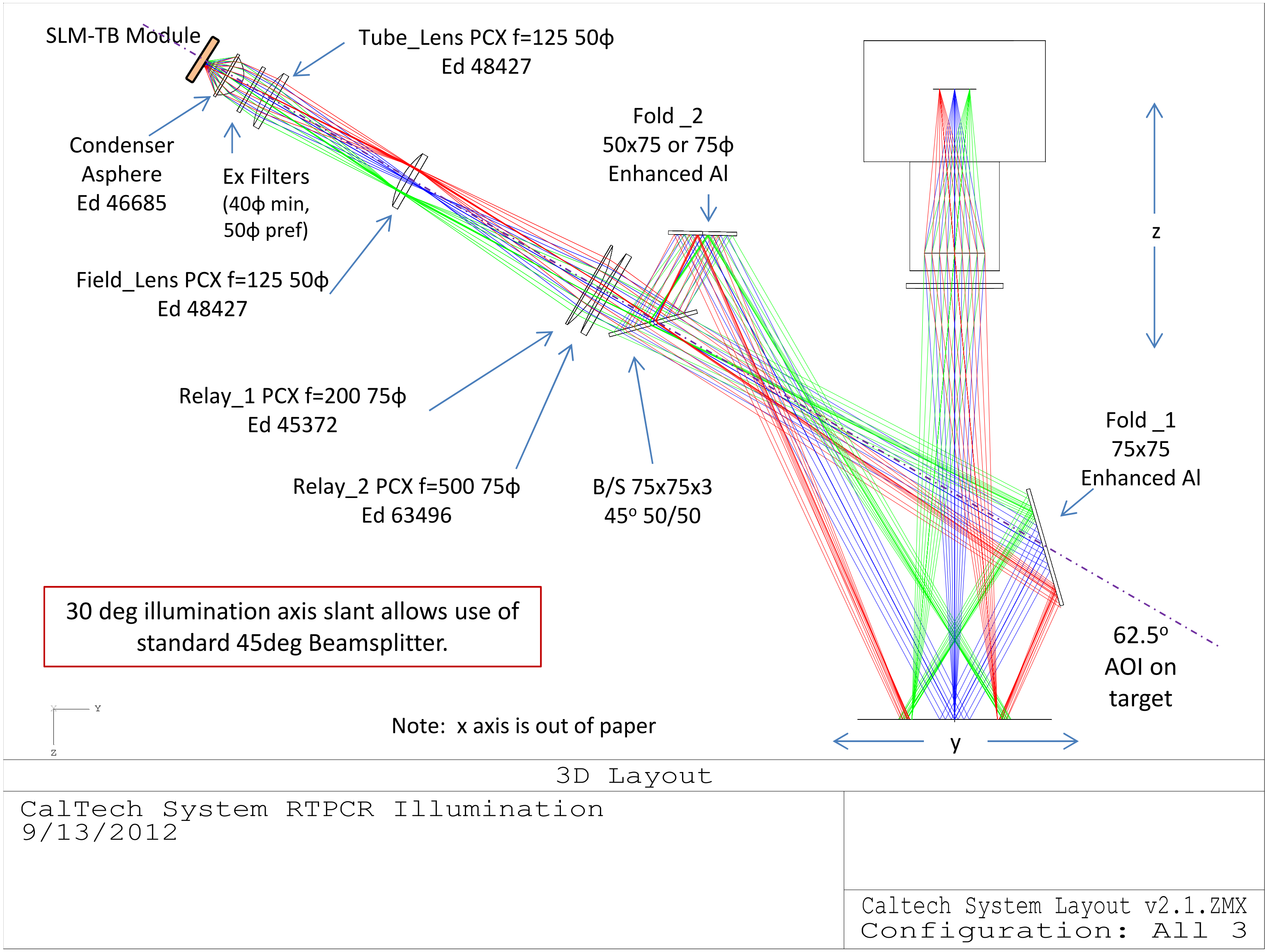

Illum System v2.1 Overall Scale

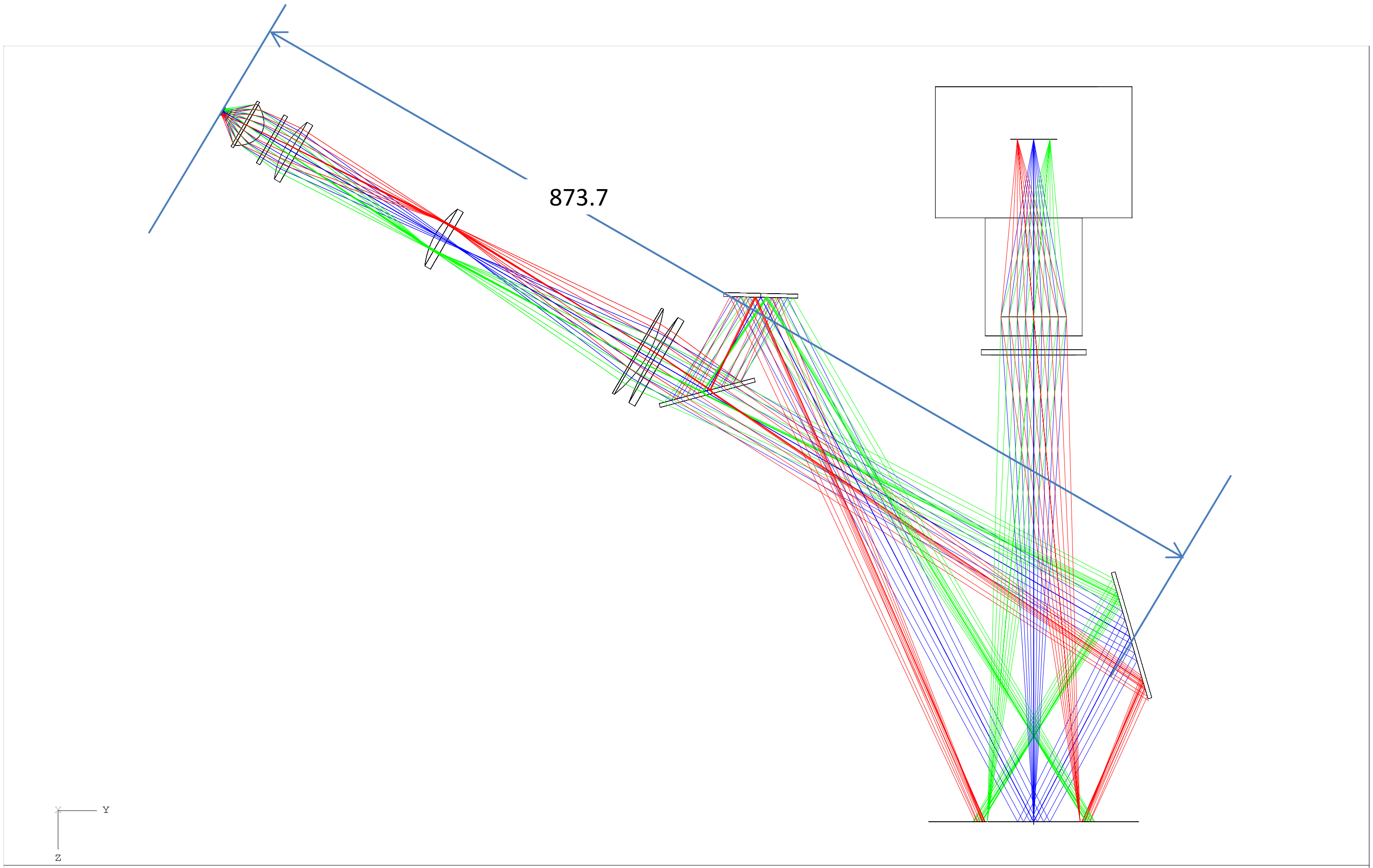

3D Layout

CalTech System RTPCR Illumination
9/13/2012

Caltech System Layout v2.1.ZMX
Configuration: All 3

Effective SLM-TB Source Radiance @ NA 0.7

(5e6 Ray Set from Philips, Non-Sequential Zemax Model)

Box is 6 mm sq

Note:
Homogeneity varies with focal plane imaged

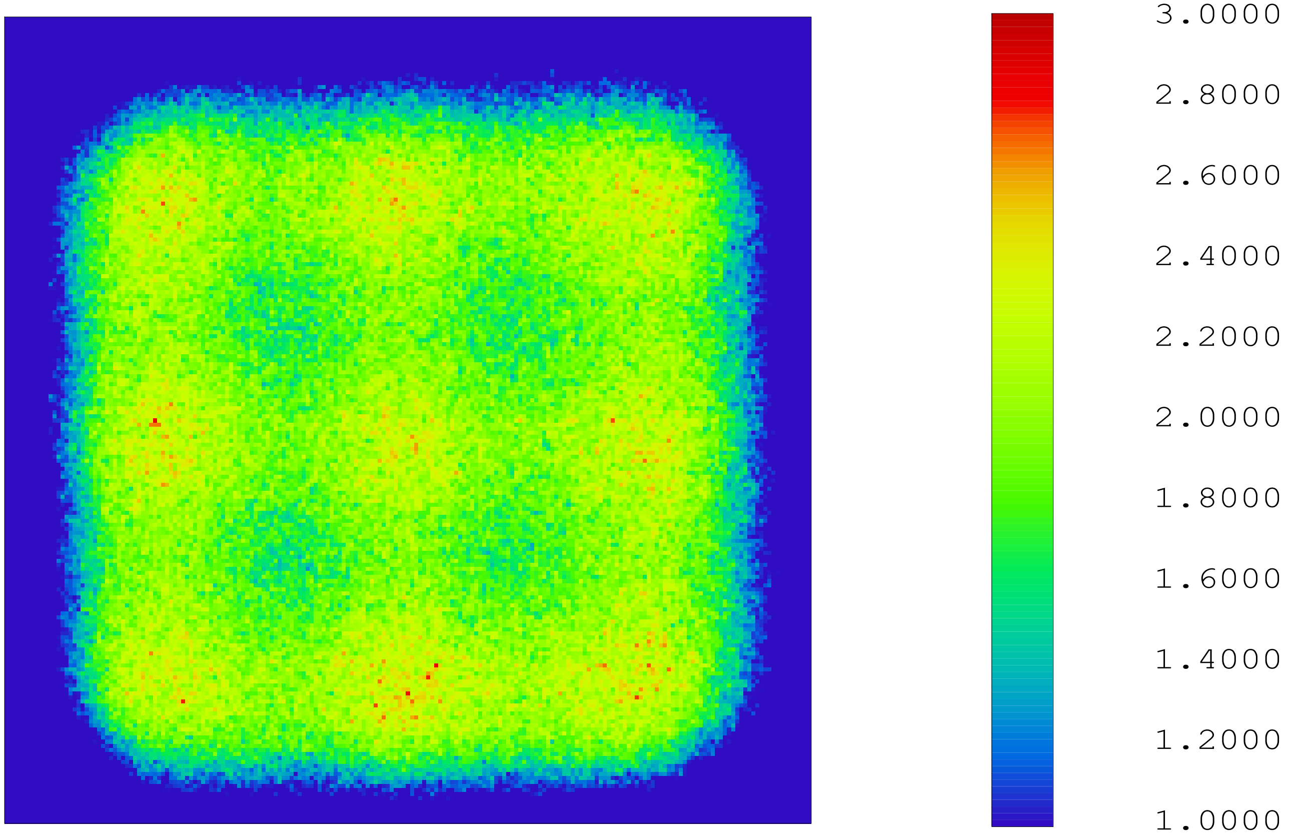

Detector Image: Irradiance

SLM-TB module source
9/12/2012
Detector 10, NSCG Surface 1:
Size 6.000 W X 6.000 H Millimeters, Pixels 201 W X 201 H, Total Hits = 2692854
Peak Irradiance : 2.832E+000 Watts/cm^2
Total Power : 5.386E-001 Watts

Calculated Dual Beam Irradiance Pattern at Target

(using 4.8 mm square approximation to SLM-TB emission)

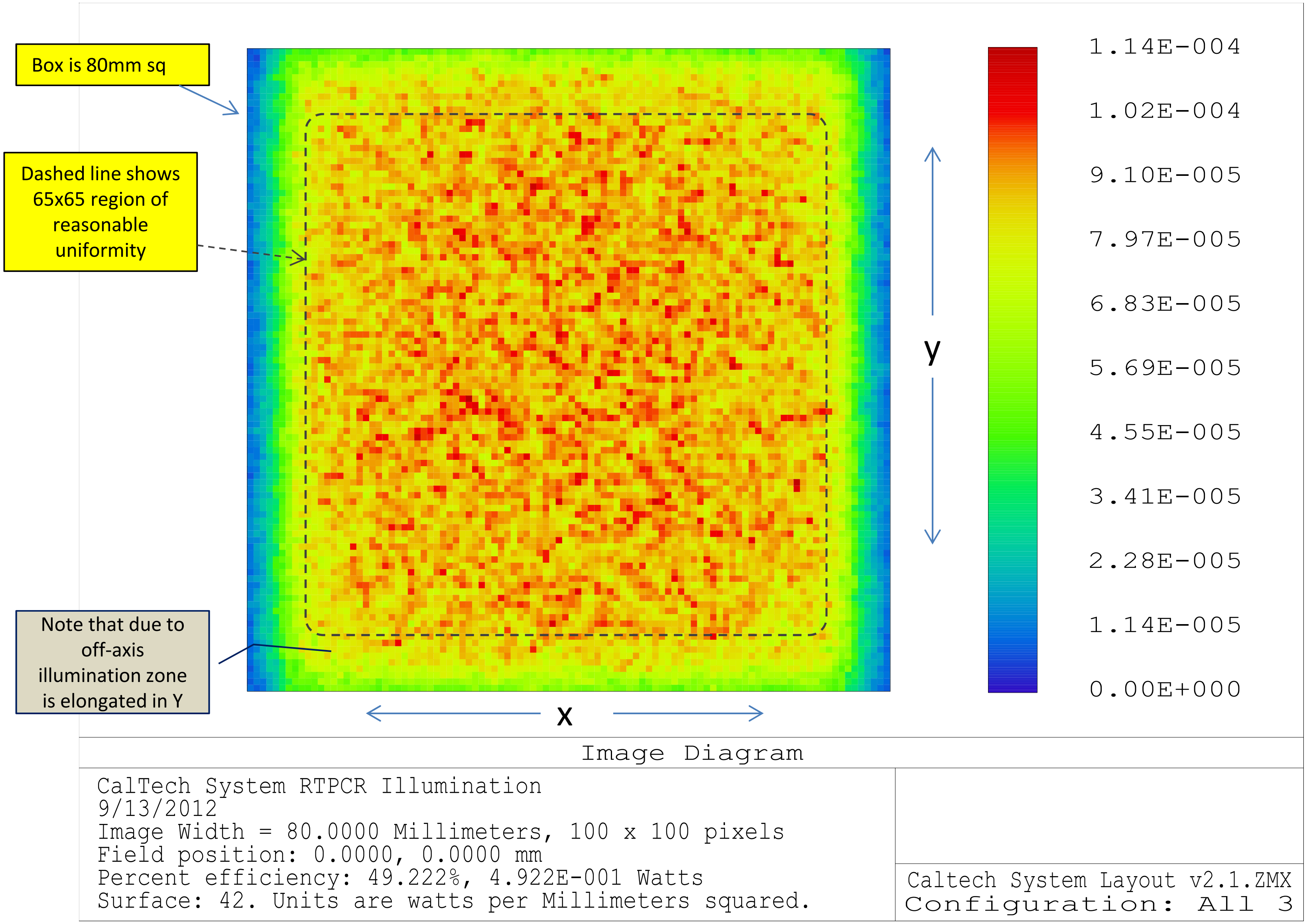

Single Beam Uniformity shows wedge

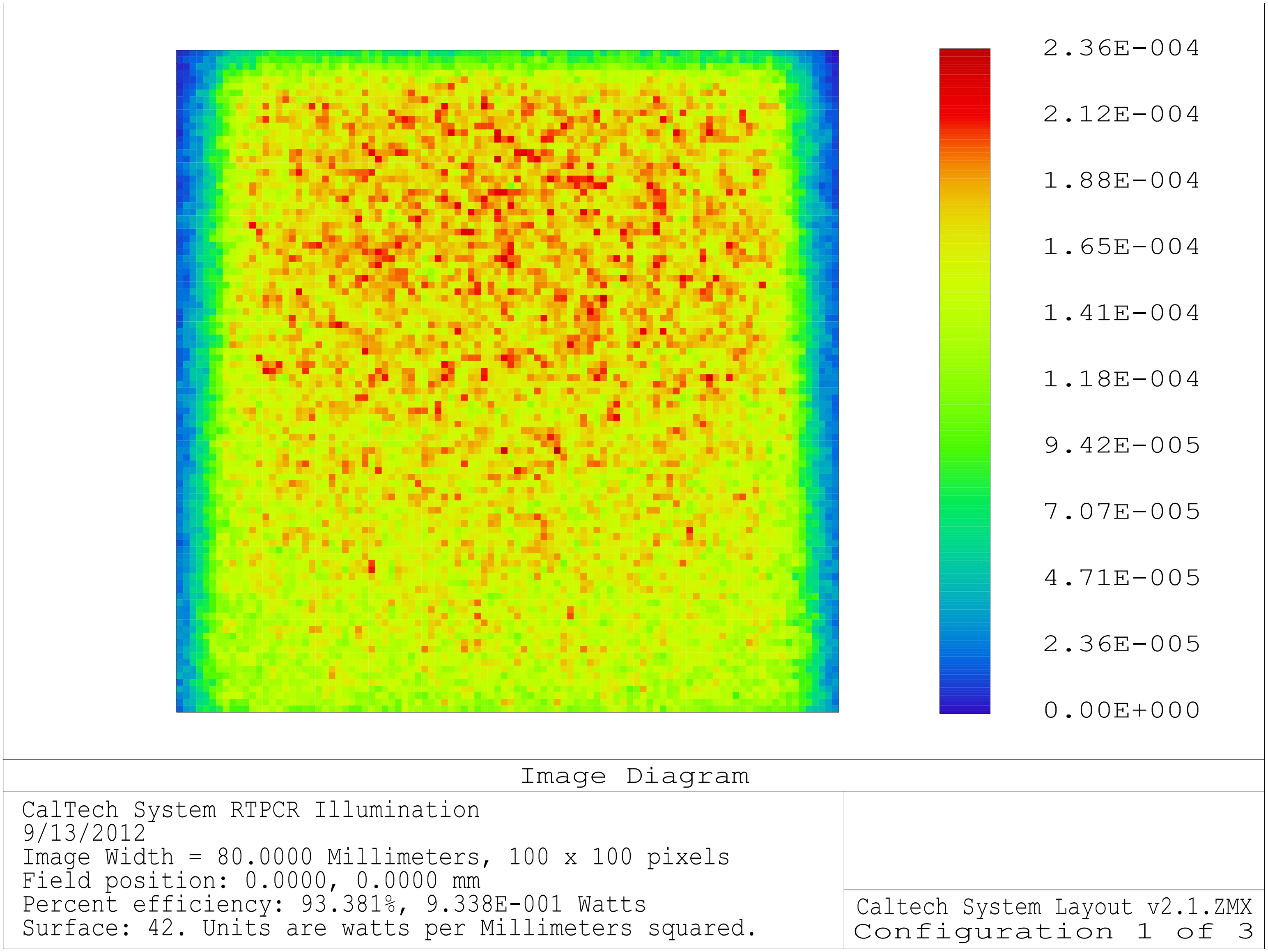

Optical Components

Optic	Description	P/N (Edmunds)	
Source	SLM-TB (or Luxeon S)		Allow ±1mm x,y, z (focus) alignment
Condenser	Fast Asphere, molded	46685	[instead focus dz here OK]
Tube and Field Lenses	BK7 PCX 125efl, 50ϕ, AR coated	48247	Lenses need only simple slide-carrier and height adjustments
Relay 1	BK7 PCX 200efl, 75ϕ	45372	
Relay 2	BK7 PCX 500efl, 75ϕ	63496	
BeamSplitter	BK7 75x75x3, 50-50 dielectric, 45 deg, random polarization	48904	Hold by +/- x edges, must be “bare” at right edge
Fold Mirror 1	Enhanced Alum, 75x100x3	48453	Need convenient tilt adjs
Fold Mirror 2	Enhanced Alum, 75x75x3	48451	Need convenient tilt adjs
Ex Filters	Minimum 40ϕ, 45ϕ better		

ALIGNMENT SECTION

Footprints

- Each image is 100x100, 75x75 or 50x50
  - This may or may not correspond to size of mirror etc.
  - If not take the smaller optic into account
- The images are shown aligned to the optic axis
  - In some cases, such as B/S, you *may* want to shift the optic slightly to better center the footprint – optically that is OK.
- These footprints are nominal
  - Allow for some variation, so try to keep clamping well away from hot regions where possible.

Excitation Filter Surf 13

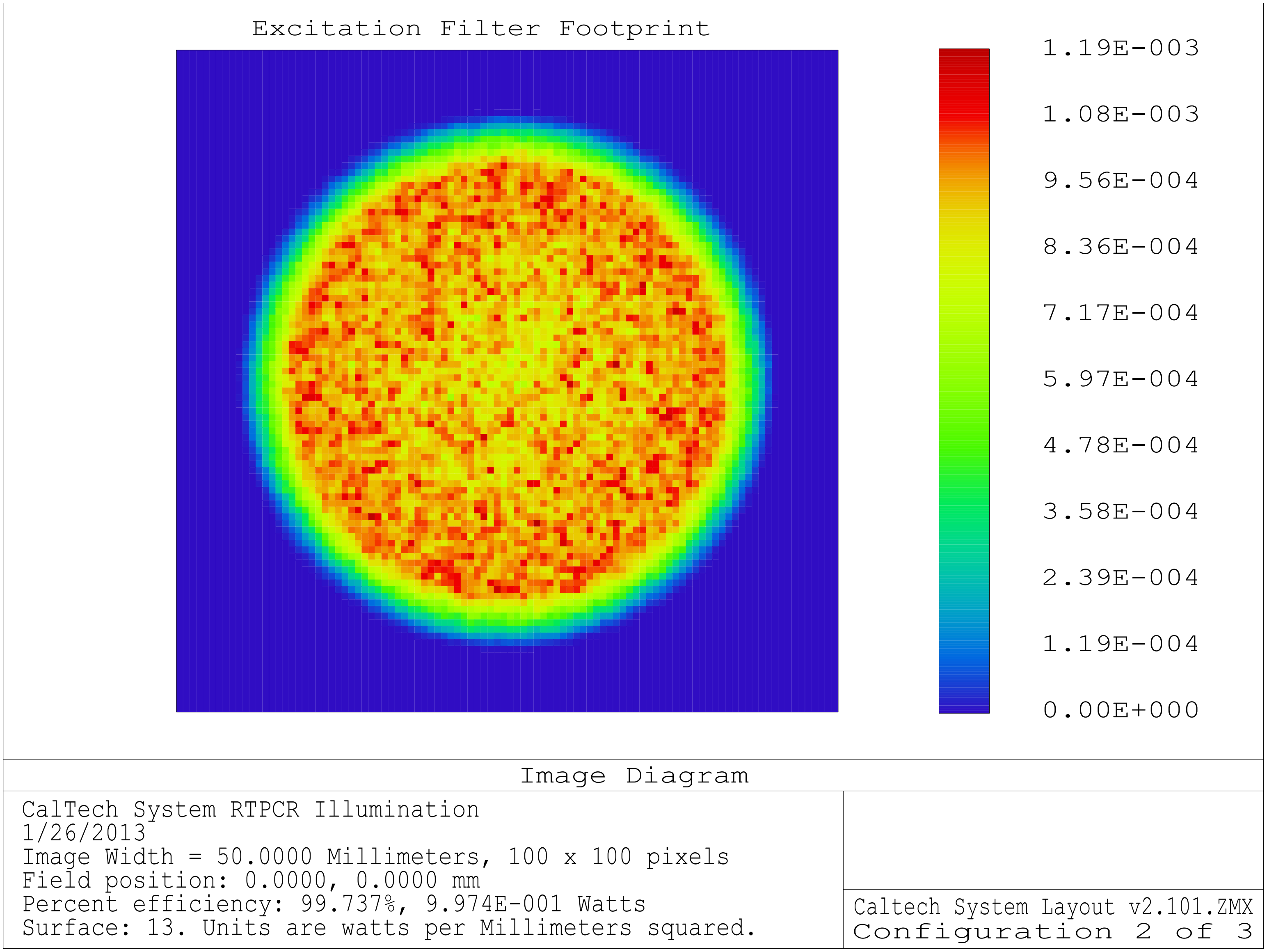

Beam Splitter (top surface)

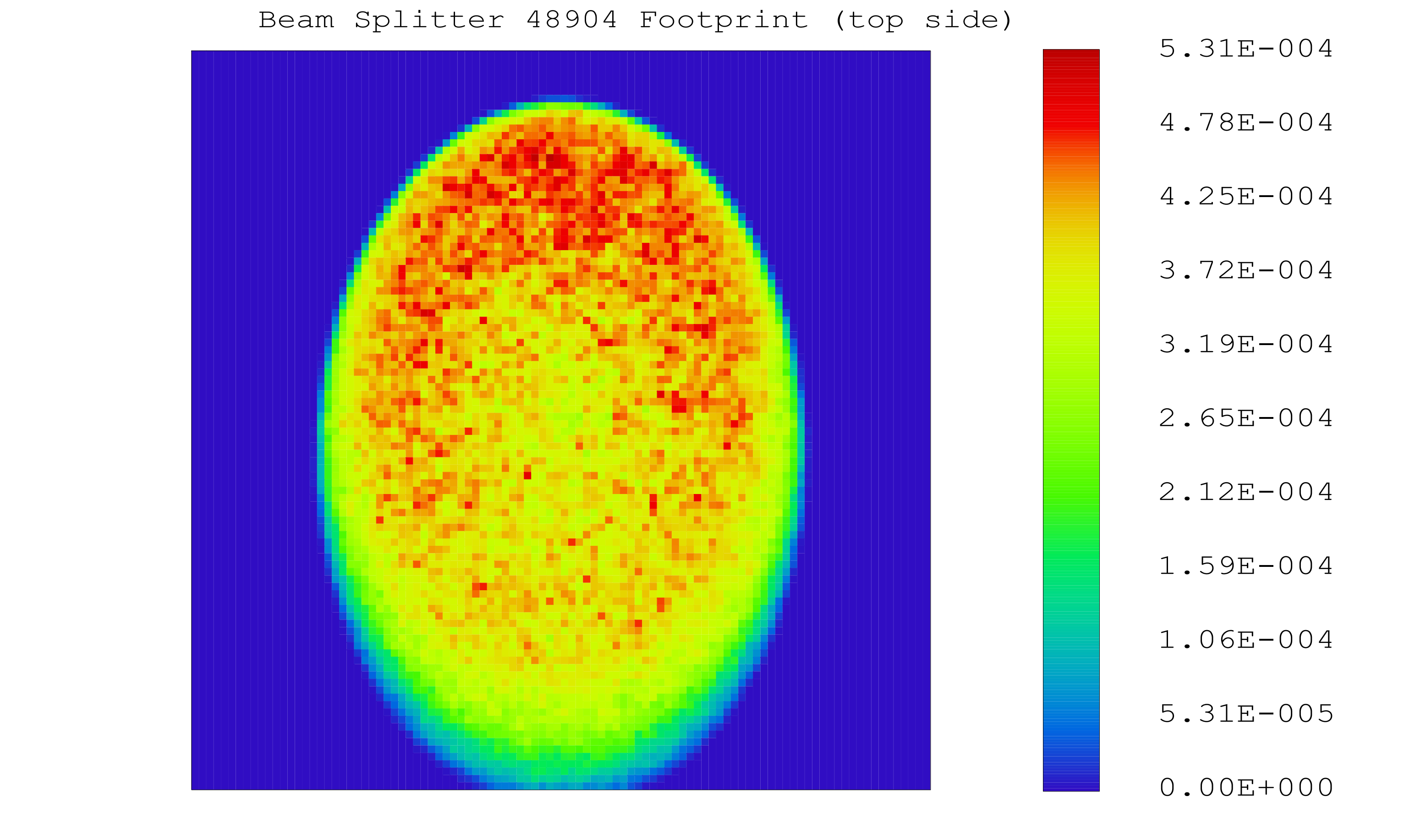

Image Diagram

CalTech System RTPCR Illumination
1/26/2013
Image Width = 75.0000 Millimeters, 100 x 100 pixels
Field position: 0.0000, 0.0000 mm
Percent efficiency: 98.039%, 9.804E-001 Watts
Surface: 27. Units are watts per Millimeters squared.

Caltech System Layout v2.101.ZMX
Configuration 2 of 3

Beam Splitter Bottom Side

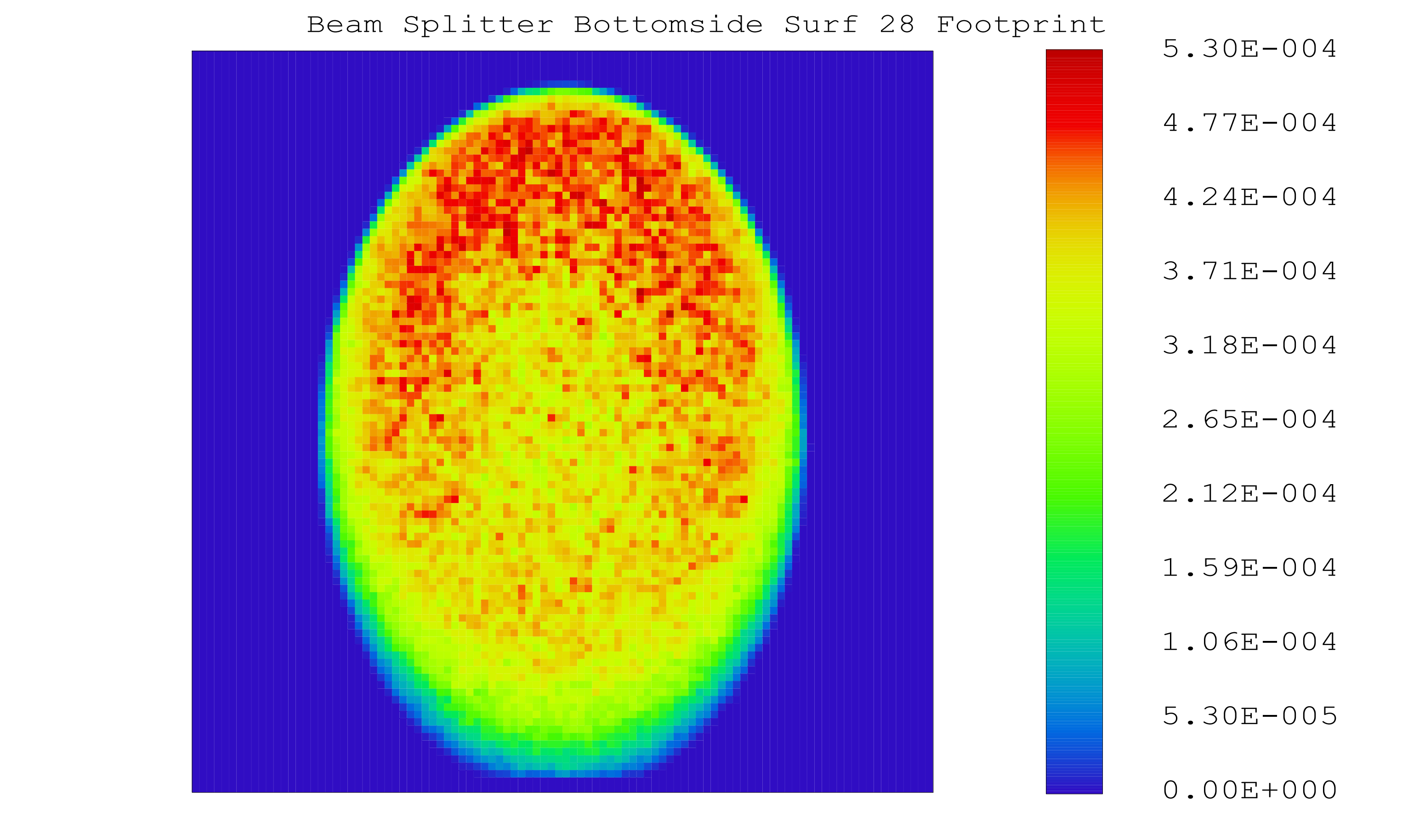

Image Diagram

CalTech System RTPCR Illumination
1/26/2013
Image Width = 75.0000 Millimeters, 100 x 100 pixels
Field position: 0.0000, 0.0000 mm
Percent efficiency: 98.045%, 9.805E-001 Watts
Surface: 28. Units are watts per Millimeters squared.

Caltech System Layout v2.101.ZMX
Configuration 1 of 3

Final Fold #1 (Surface 31)

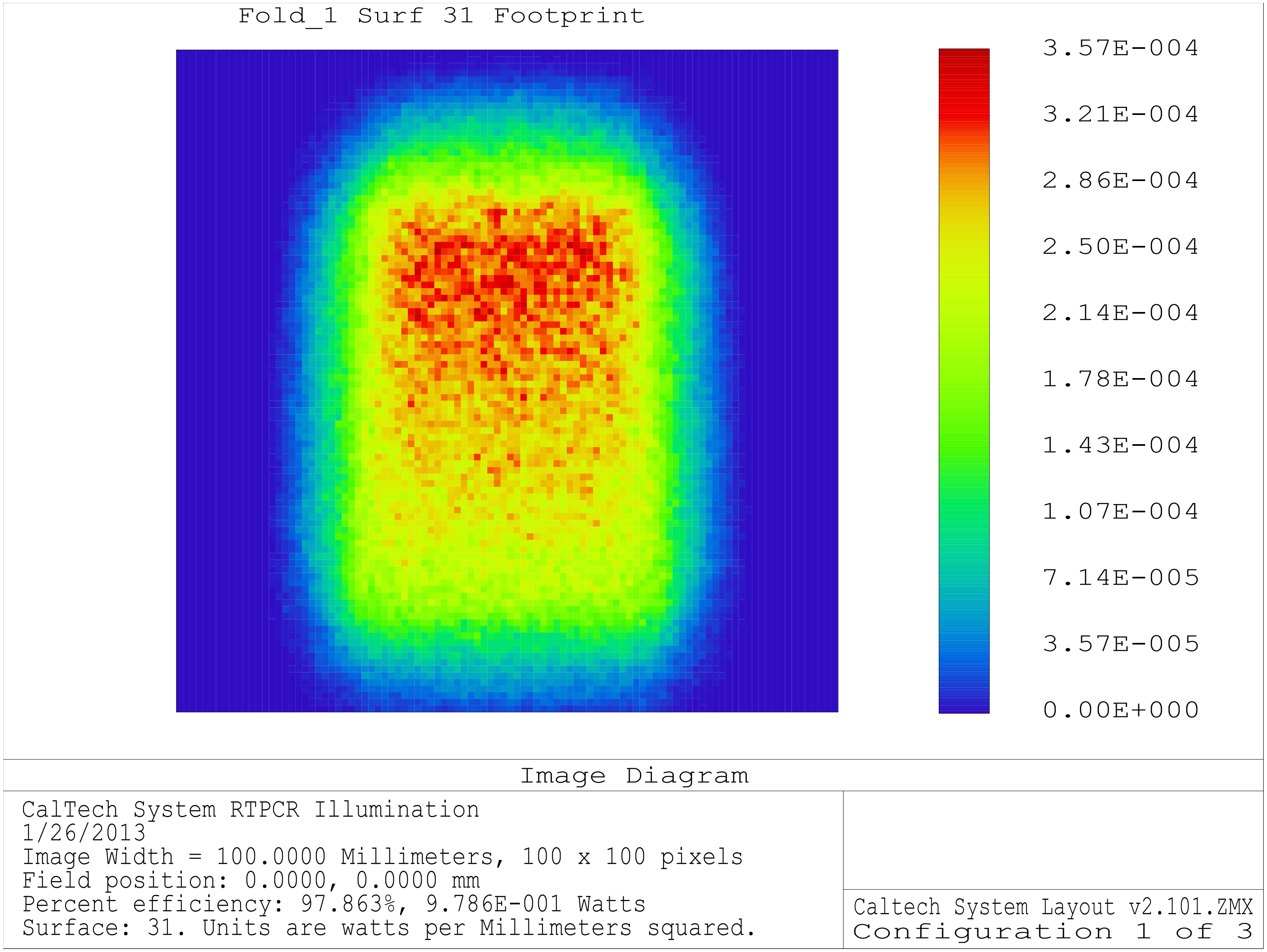

Final Fold #2 (kinked path, Surf 38)

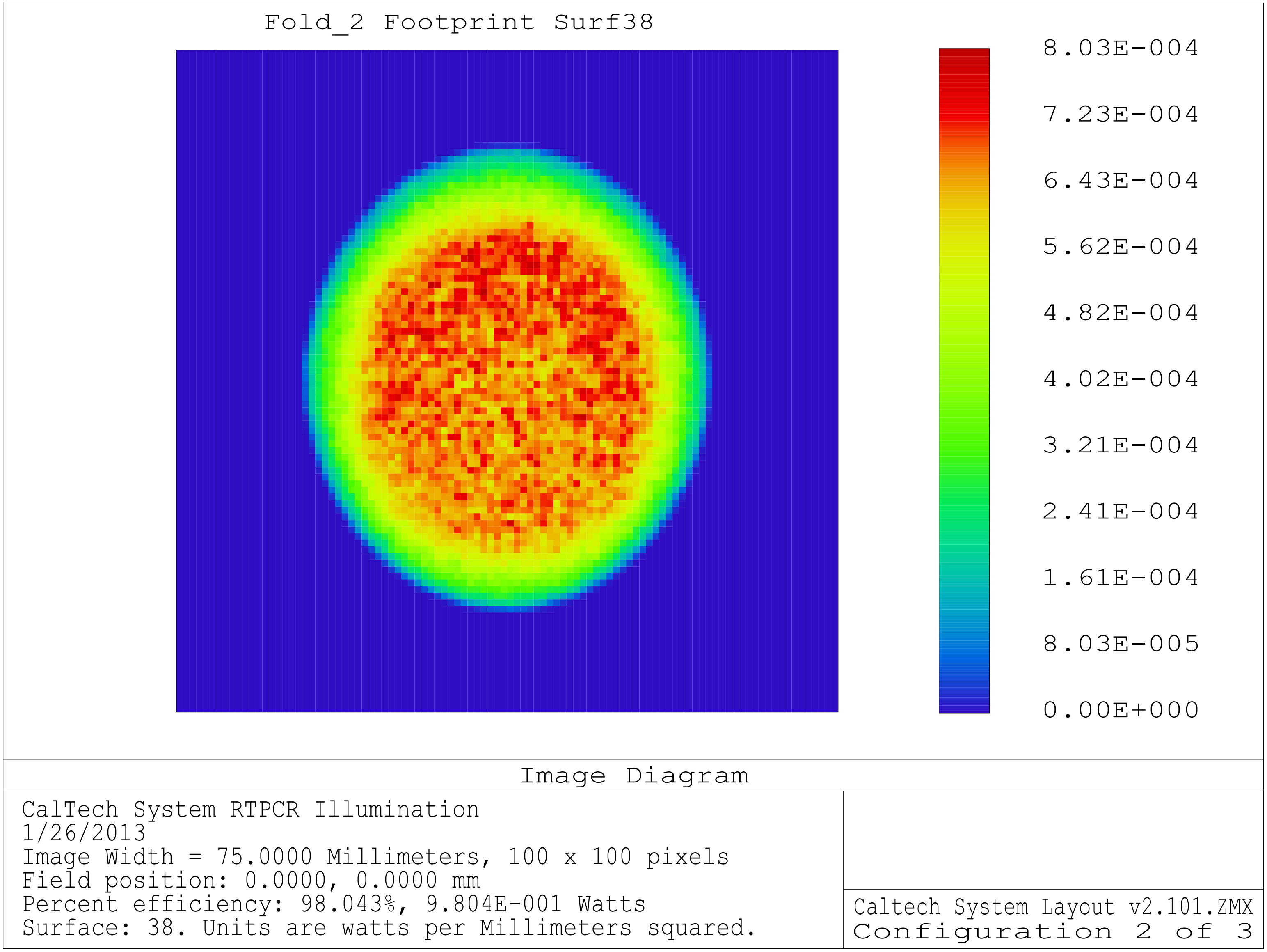

Alignment Tool

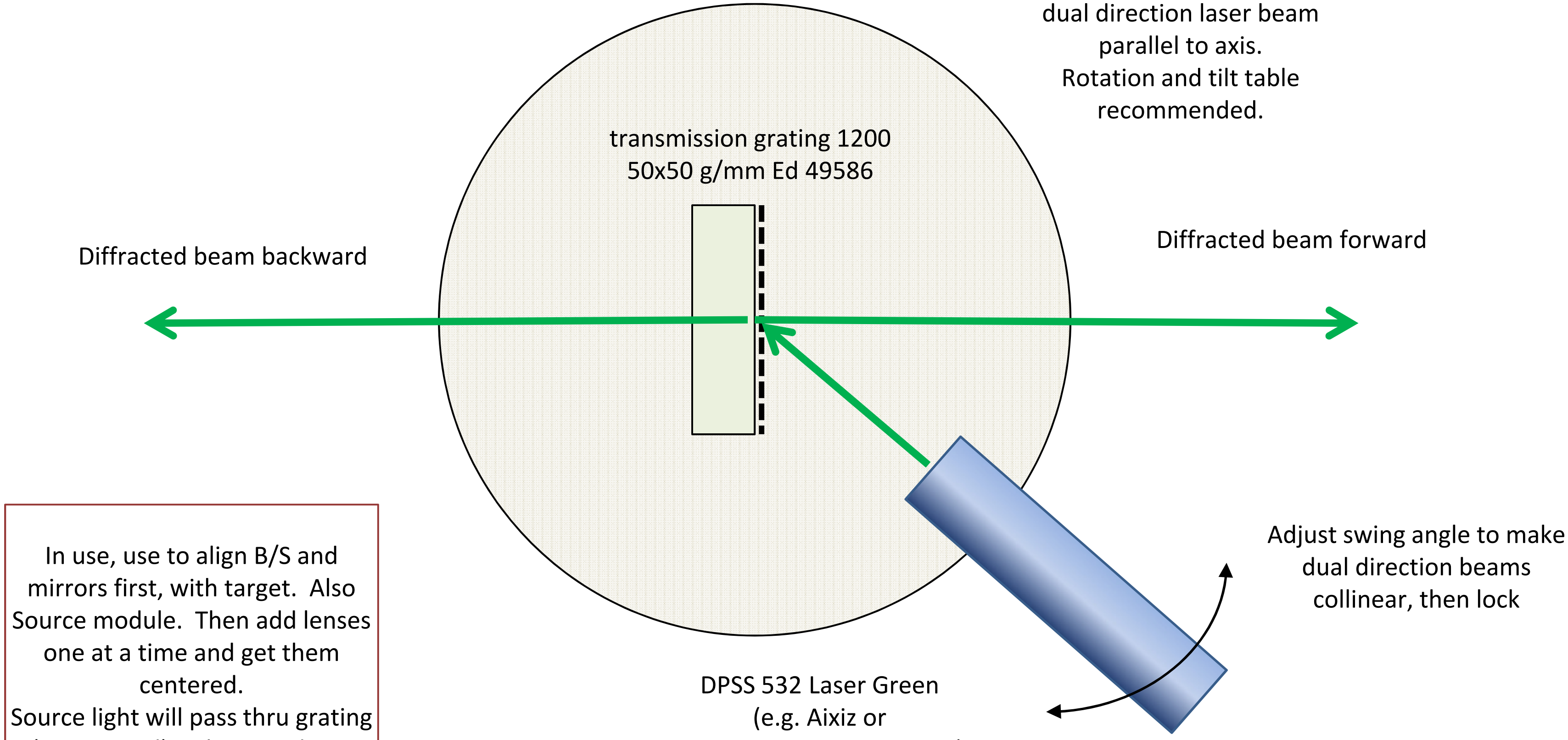

In use, use to align B/S and mirrors first, with target. Also Source module. Then add lenses one at a time and get them centered.
Source light will pass thru grating (attenuated) so laser and LED module can be simultaneously on for alignment.

Step 1: Flat Optics

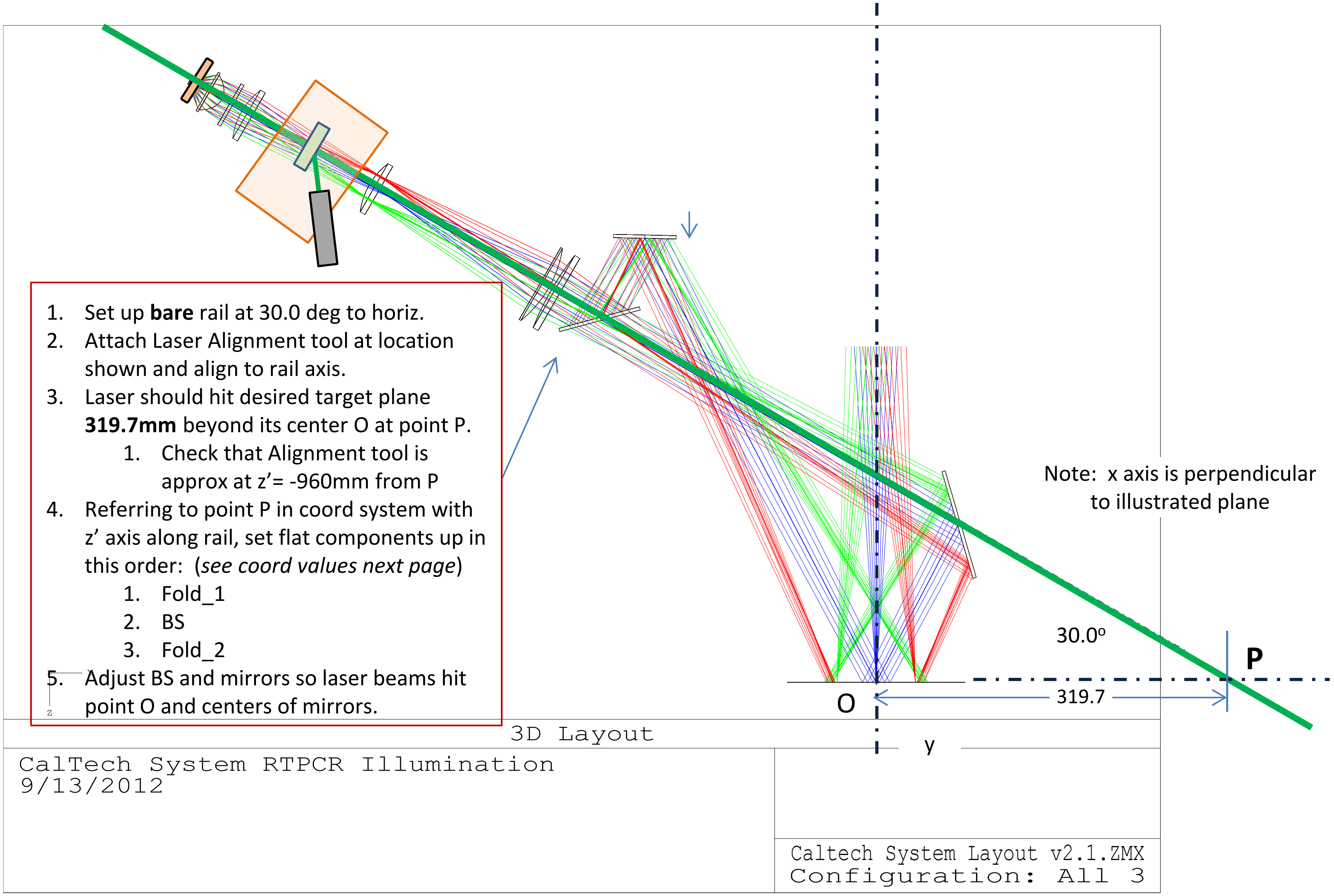

Step 1: Align Flats using primed coordinates aligned along rail

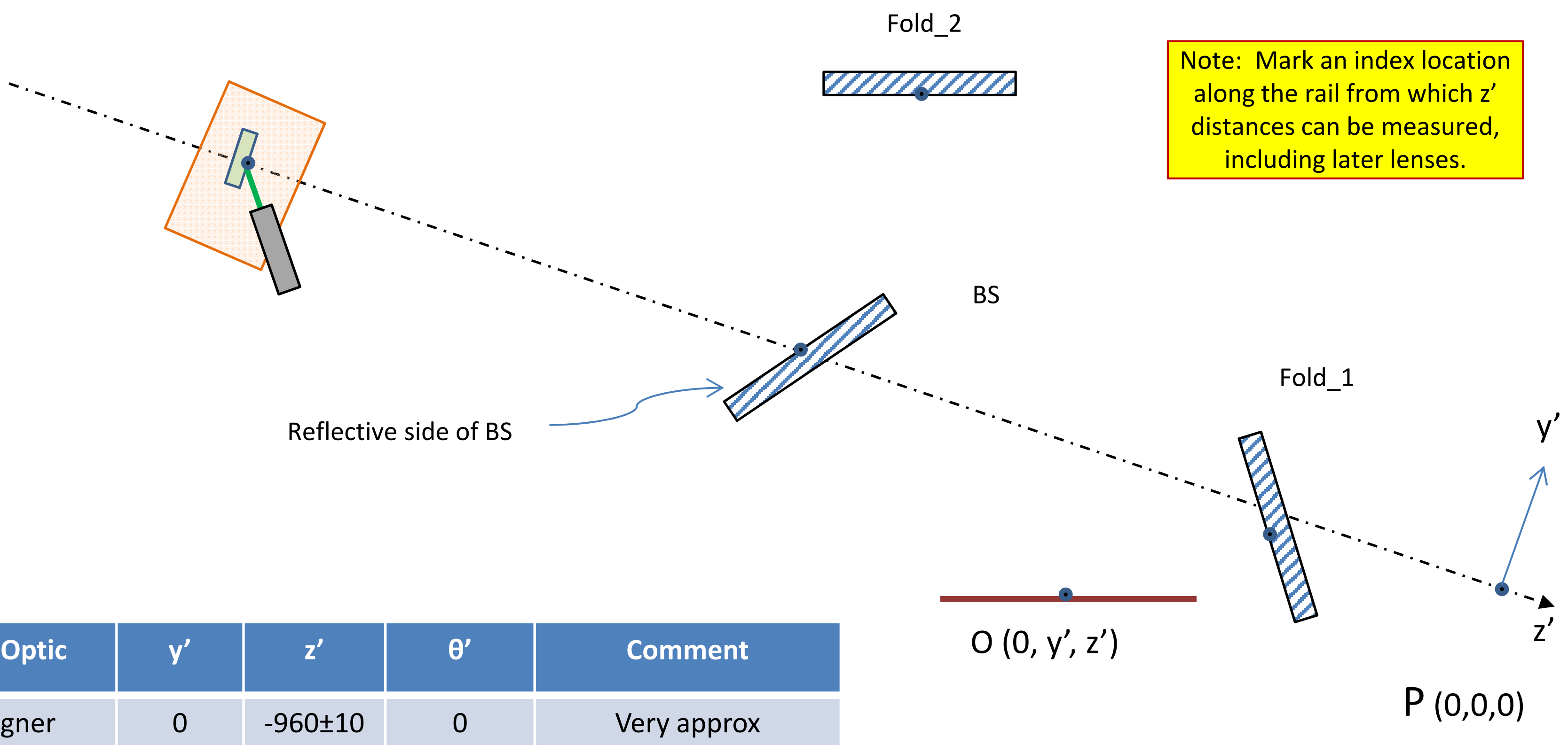

Note: Mark an index location along the rail from which z’ distances can be measured, including later lenses.

Optic	y’	z’	θ’	Comment
Aligner	0	-960±10	0	Very approx
Fold_2	82.4	-657.1	120	Horizontal
BS	0	-657.1	45	
Fold_1	-0.50	-284.3	46.25	slight y offset due to refraction in BS
Target O	-159.8	-276.9	60	Horizontal (for ref.)

Try to get to ~ 1-2mm initially, will save trouble later.

It is recommended **not** to adjust axial positions of Flats later on – leave them at design values.

Step 2: Lenses

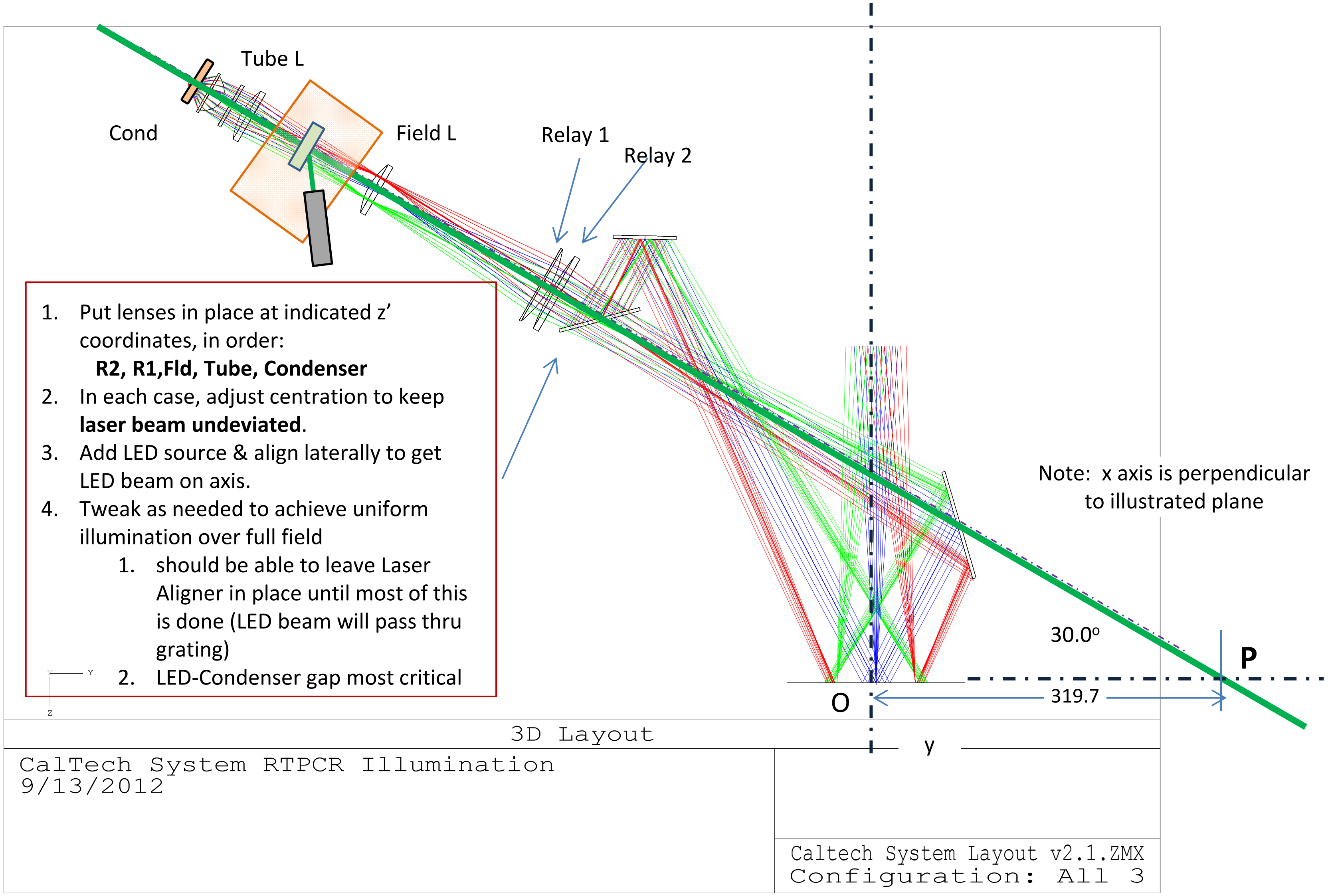

Lens Coordinates in Rail System Relative to P

(Lens Vertices to left, first encountered by light)

Lens	x’, y’	z’	Orientation	Comment
Relay 2	0	-706.8	Vertex on convex side	
Relay 1	0	-718.5	Vertex on flat side	
Field	0	-896.0	Vertex on convex side	
Tube	0	-1028.5	Vertex on convex side	
Condenser	0	-1064.5	Vertex on Flat Side	
Source	0	-1083		Effective src location, adj as needed

Try to get to ~ 1-2mm initially, will save trouble later.

Mark initial positions so that if you play with alignment and “walk away” you can get back to nominal settings easily.

Further Alignment Steps

- Add Excitation Filter Wheel
- Add Camera Column
  - Find height(s) at which desired magnification(s) are achieved.
- Add Emission Filter Wheel
