# Supplementary material for: Instrument for Real-Time Digital Nucleic Acid Amplification on Custom Microfluidic Devices
Source: PLoS One. 2016 Oct 19;11(10):e0163060. doi: 10.1371/journal.pone.0163060 (PMC5070811; doi:10.1371/journal.pone.0163060)
Supplement: S2 File — A detailed list of all components used in construction of the real-time digital instrument. (PDF) [file pone.0163060.s002.pdf]

S2. Detailed parts list. A detailed list of all components used in construction of the real-time digital instrument.

*All prices and websites are current as of April 2014					
**Part Number**	**Supplier**	**Price**	**Quantity**	**Website**	**Comments**
VX-29MG-M2-A0-F-2	Vision Systems Technology	Quote	1	visionsystech.com	
Zeiss Makro-Planar T* 100mm f/2 ZF.2	B&H Photo Video	$ 1,995.00	1	bhphotovideo.com	
CFW-1-5	Finger Lakes International	$ 895.00	1	flicamera.com	
CFW-6-6	Finger Lakes International	$ 1,995.00	1	flicamera.com	
XF3406 79mm	Omega Optical	Custom	1	omegafilters.com	In the original instrument two different suppliers of filters were used.
XF3402 79mm	Omega Optical	Custom	1	omegafilters.com	Shown here are filters of equivalent quality from a single supplier.
XF3409 79mm	Omega Optical	Custom	1	omegafilters.com	
XF1411 50.8mm	Omega Optical	Custom	1	omegafilters.com	
XF1413 50.8mm	Omega Optical	Custom	1	omegafilters.com	
XF1414 50.8mm	Omega Optical	Custom	1	omegafilters.com	
LXS8-PW40	Future Lighting Solutions	$ 13.25	2	futurelightingsolutions.com	
913701213402	Future Lighting Solutions	$ 37.38	1	futurelightingsolutions.com	
HSLCS-CALCL-007	Digi-Key	$ 13.37	1	http://www.digikey.com/product-search/en?x=0&y=0&lang=en&site=us&KeyWords=HSLCS-CALCL-007	
SSLCS-CM012-002	Digi-Key	$ 23.12	1	http://www.digikey.com/product-search/en?vendor=0&keywords=SSLCS-CM012-002	
WALLS-C4600-001	Digi-Key	$ 2.30	1	http://www.digikey.com/product-search/en?vendor=0&keywords=WALLS-C4600-001	
2154235-1	Digi-Key	$ 3.04	2	http://www.digikey.com/product-search/en?vendor=0&keywords=2154235-1	
615-1069-ND	Digi-Key	$ 8.12	3	http://www.digikey.com/product-search/en?x=18&y=18&lang=en&site=us&KeyWords=615-1069-ND	
926-1008-ND	Digi-Key	$ 7.43	1	http://www.digikey.com/product-search/en?x=0&y=0&lang=en&site=us&KeyWords=926-1008-ND	
926-1157-ND	Digi-Key	$ 25.73	1	http://www.digikey.com/product-search/en?vendor=0&keywords=926-1157-ND	
80-2-5-ND	Digi-Key	$ 4.38	1	http://www.digikey.com/product-search/en?vendor=0&keywords=80-2-5-ND	
HR1587-ND	Digi-Key	$ 11.16	1	http://www.digikey.com/product-search/en?x=0&y=0&lang=en&site=us&KeyWords=%09+HR1587-ND	

335-1155-ND	Digi-Key	$ 0.55	6	http://www.digikey.com/product-search/en?x=0&y=0&lang=en&site=us&KeyWords=335-1155-ND	
04UTS205	Melles Griot	$ 803.00	1	http://marketplace.idexop.com/store/IdexCustom/PartDetails?pvId=35918	
04ISC850	Melles Griot	$ 903.00	1	http://marketplace.idexop.com/store/IdexCustom/PartDetails?pvId=35866	
04IPS833	Melles Griot	$ 79.00	1	http://marketplace.idexop.com/store/IdexCustom/PartDetails?pvId=35863	
OMB-DAQ-2408-2AO	Omega Engineering	$ 699.00	1	http://www.omega.com/pptst/OMB-DAQ-2408.html	
5TC-TT-K-36-36	Omega Engineering	$ 53.00	1	http://www.omega.com/pptst/5TC.html	
C01-K	Omega Engineering	$ 63.00	1	http://www.omega.com/pptst/CO-K.html	
PTC-200 Thermocycler	Bio-Rad	Used	1	Various places	
NT46-685	Edmund Optics	$ 45.00	1	http://www.edmundoptics.com/optics/optical-lenses/aspheric-lenses/aspheric-condenser-lenses/46-685	
NT48-247	Edmund Optics	$ 50.00	2	http://www.edmundoptics.com/optics/optical-lenses/plano-convex-pcx-spherical-singlet-lenses/vis-0-coated-plano-convex-pcx-lenses/48-247	
NT45-372	Edmund Optics	$ 60.00	1	http://www.edmundoptics.com/optics/optical-lenses/plano-convex-pcx-spherical-singlet-lenses/mgf2-coated-plano-convex-pcx-lenses/45-372	
NT63-496	Edmund Optics	$ 60.00	1	http://www.edmundoptics.com/optics/optical-lenses/plano-convex-pcx-spherical-singlet-lenses/mgf2-coated-plano-convex-pcx-lenses/63-496	
NT48-904	Edmund Optics	$ 215.00	1	http://www.edmundoptics.com/optics/beamsplitters/plate-beamsplitters/visible-nir-plate-beamsplitters/48-904	
NT48-453	Edmund Optics	$ 31.50	1	http://www.edmundoptics.com/optics/optical-mirrors/flat-mirrors/first-surface-mirrors/48-453	
NT48-452	Edmund Optics	$ 30.00	1	http://www.edmundoptics.com/optics/optical-mirrors/flat-mirrors/first-surface-mirrors/48-452	
64-566	Edmund Optics	$ 50.00	1	http://www.edmundoptics.com/optomechanics/optical-mounts-plates/lens-mounts/optic-component-mounts/64-566	
64-567	Edmund Optics	$ 50.00	1	http://www.edmundoptics.com/optomechanics/optical-mounts-plates/lens-mounts/optic-component-mounts/64-567	
44N588	Grainger	$ 95.45	1	http://www.grainger.com/product/SINTRA-PVC-Board-44N588?searchQuery=44N588	
2GXZ4	Grainger	$ 13.71	1	http://www.grainger.com/product/PERMATEX-RTV-Silicone-2GXZ4?s_pp=false	
2GXY5	Grainger	$ 11.43	1	http://www.grainger.com/product/PERMATEX-RTV-Silicone-2GXY5?functionCode=P2IDP2PCP	
N82E16835186020	Newegg	$ 7.99	1	http://www.newegg.com/Product/Product.aspx?Item=N82E16835186020	
18AWG Wire	Amazon	$ 15.99	1	http://www.amazon.com/Menotek-Speaker-Spool-18AWG-Installation/dp/B00DILPVIO/ref=zg_bs_464416_81	
BNC Connector	Amazon	$ 8.99	1	http://www.amazon.com/Bluecell-Coaxial-Camera-Vedio-Connector/dp/B00EB3HYWE/ref=sr_1_21?s=electronics&ie=UTF8&qid=1393655896&sr=1-21&keywords=bnc+connector	

Solder	Amazon	$ 6.50	1	http://www.amazon.com/Veecome-0-6MM-Diameter-Solder-Soldering/dp/B007KIGO8M/ref=zg_bs_13837421_2	
1010-Black x 48 N	EBAY (80/20 inc)	$ 15.87	16	http://www.ebay.com/itm/8020-T-Slot-Aluminum-Extrusion-10-S-1010-Black-x-48-N-/330759984812?pt=LH_DefaultDomain_0&hash=item4d02d4d6ac	
4151 N	EBAY (80/20 inc)	$ 6.30	18	http://www.ebay.com/itm/80-20-T-Slot-Aluminum-Joining-Plate-10-S-4151-N-/330343501054?pt=LH_DefaultDomain_0&hash=item4cea01ccfe	
4081 N	EBAY (80/20 inc)	$ 6.55	2	http://www.ebay.com/itm/8020-T-Slot-Aluminum-Joining-Plate-10-S-4081-N-/370137832668?pt=LH_DefaultDomain_0&hash=item562def1cdc	
4080 N	EBAY (80/20 inc)	$ 7.00	2	http://www.ebay.com/itm/8020-T-Slot-Aluminum-5-Hole-T-Joining-Plate-10-Series-4080-N-/221074562445?pt=LH_DefaultDomain_0&hash=item3379121d8d	
2116 N	EBAY (80/20 inc)	$ 1.11	40	http://www.ebay.com/itm/80-20-T-Slot-10-Series-Rubber-Panel-Gasket-2116-N-/370135230407?pt=LH_DefaultDomain_0&hash=item562dc767c7	
2838 N	EBAY (80/20 inc)	$ 6.15	3	http://www.ebay.com/itm/80-20-Inc-10-Series-Right-Hand-Long-Pin-Economy-Lift-Off-Hinge-2838-N-/370668837355?pt=LH_DefaultDomain_0&hash=item564d9599eb	
2053 N	EBAY (80/20 inc)	$ 18.90	1	http://www.ebay.com/itm/8020-Inc-Deadbolt-w-Top-Latch-10-S-15-S-2053-N-/220339939313?pt=LH_DefaultDomain_0&hash=item334d48a7f1	
2062 N	EBAY (80/20 inc)	$ 4.20	1	http://www.ebay.com/itm/8020-T-Slot-Small-Plastic-Door-Handle-10-15-S-2062-N-/220705273707?pt=LH_DefaultDomain_0&hash=item33630f376b	
3382 N	EBAY (80/20 inc)	$ 5.25	6	http://www.ebay.com/itm/8020-T-Slot-Hardware-1-4-20-Economy-T-Nut-10-Series-3382-25-Pcs-N-/330861775900?pt=LH_DefaultDomain_0&hash=item4d08e60c1c	
MB60120/M	Thorlabs	$ 923.00	1	thorlabs.com	
XT95-200	Thorlabs	Custom	1	thorlabs.com	
XT95-300	Thorlabs	Custom	1	thorlabs.com	
XT95-500	Thorlabs	$ 140.00	1	thorlabs.com	
XT95-1000	Thorlabs	$ 210.00	1	thorlabs.com	
XT66-500	Thorlabs	$ 79.56	1	thorlabs.com	
XT66-100	Thorlabs	$ 32.64	4	thorlabs.com	
XT95P4	Thorlabs	$ 98.00	2	thorlabs.com	
XT95P11/M	Thorlabs	$ 77.70	3	thorlabs.com	
XT95P3	Thorlabs	$ 48.00	3	thorlabs.com	
XT95P12/M	Thorlabs	$ 43.05	4	thorlabs.com	
XT95A66	Thorlabs	$ 62.00	4	thorlabs.com	
XT66C4	Thorlabs	$ 26.52	6	thorlabs.com	
SFH2	Thorlabs	$ 65.10	6	thorlabs.com	
PJ301/M	Thorlabs	$ 16.00	1	thorlabs.com	

BA1S/M	Thorlabs	$ 5.20	1	thorlabs.com	
BA1/M	Thorlabs	$ 5.60	4	thorlabs.com	
RA90/M	Thorlabs	$ 9.48	13	thorlabs.com	
AP4M3M	Thorlabs	$ 1.90	6	thorlabs.com	
MS1.5R/M	Thorlabs	$ 6.10	6	thorlabs.com	
RA180/M	Thorlabs	$ 10.00	6	thorlabs.com	
SWC/M	Thorlabs	$ 21.90	2	thorlabs.com	
LMR75/M	Thorlabs	$ 51.45	2	thorlabs.com	
XT66P1	Thorlabs	$ 34.68	1	thorlabs.com	
BLP01/M	Thorlabs	$ 94.35	2	thorlabs.com	
SS25E63D	Thorlabs	$ 11.00	1	thorlabs.com	
SS6M16D	Thorlabs	$ 11.00	1	thorlabs.com	
TR150/M	Thorlabs	$ 6.77	2	thorlabs.com	
TR75/M	Thorlabs	$ 5.42	9	thorlabs.com	
TR250/M	Thorlabs	$ 9.02	6	thorlabs.com	
TR30/M	Thorlabs	$ 4.74	7	thorlabs.com	
TR50/M	Thorlabs	$ 5.19	7	thorlabs.com	
TR40/M	Thorlabs	$ 4.97	2	thorlabs.com	
TR3	Thorlabs	$ 5.42	2	thorlabs.com	
TR20/M	Thorlabs	$ 4.74	1	thorlabs.com	
PH40/M	Thorlabs	$ 7.22	7	thorlabs.com	
PH75/M	Thorlabs	$ 8.27	2	thorlabs.com	
PH50/M	Thorlabs	$ 7.70	2	thorlabs.com	
92311A199	McMaster Carr	$ 8.11	1	mcmaster.com	
91292A135	McMaster Carr	$ 7.07	1	mcmaster.com	
91292A137	McMaster Carr	$ 4.31	1	mcmaster.com	
91292A121	McMaster Carr	$ 8.00	1	mcmaster.com	
91292A117	McMaster Carr	$ 6.40	1	mcmaster.com	
91292A134	McMaster Carr	$ 7.22	1	mcmaster.com	
93475A230	McMaster Carr	$ 1.86	1	mcmaster.com	
93475A250	McMaster Carr	$ 4.86	1	mcmaster.com	
91296A190	McMaster Carr	$ 5.00	1	mcmaster.com	
92196A537	McMaster Carr	$ 7.31	3	mcmaster.com	
92141A029	McMaster Carr	$ 3.37	2	mcmaster.com	

92805K13	McMaster Carr	$ 2.47	1	mcmaster.com	
58-985	Edmund Optics	$ 5.80	3	http://www.edmundoptics.com/optomechanics/optical-breadboard-components-laboratory-tables/posts-post-holders/thread-adapters/2787	
64-567	Edmund Optics	$ 50.00	1	http://www.edmundoptics.com/optomechanics/optical-mounts-plates/lens-mounts/optic-component-mounts/64-567	
PL2303HX Rev D	Amazon	$ 14.95	1	http://www.amazon.com/Plugable-Adapter-Prolific-PL2303HX-Chipset/dp/B00425S1H8/ref=sr_1_2?ie=UTF8&qid=1394562702&sr=8-2&keywords=rs232+to+usb	
1010-Black x 48 N	EBAY (80/20 inc)	$ 15.87	3	http://www.ebay.com/itm/8020-T-Slot-Aluminum-Extrusion-10-S-1010-Black-x-48-N-/330759984812?pt=LH_DefaultDomain_0&hash=item4d02d4d6ac	
1020-Black x 48 N	Ebay	$ 24.51	3	http://www.ebay.com/itm/80-20-T-Slot-Aluminum-Extrusion-10-S-1020-Black-x-48-N-/221068427368?pt=LH_DefaultDomain_0&hash=item3378b48068	
2192 N	Ebay	$ 12.10	4	http://www.ebay.com/itm/8020-T-Slot-Deluxe-1-4-20-Leveling-Foot-10-S-2192-N-/220705273710?pt=LH_DefaultDomain_0&hash=item33630f376e	
1010 x 36 Black	Ebay	$ 9.40	4	http://www.ebay.com/itm/80-20-Inc-1-x-1-T-Slot-Aluminum-Extrusion-10-Series-1010-x-36-Black-/191131279268?pt=LH_DefaultDomain_0&hash=item2c804fdfa4	
4150 N	Ebay	$ 6.80	8	http://www.ebay.com/itm/8020-T-Slot-Aluminum-Joining-Plate-10-S-4140-N-/330295191719?pt=LH_DefaultDomain_0&hash=item4ce720a8a7	
4151 N	Ebay	$ 6.30	16	http://www.ebay.com/itm/80-20-T-Slot-Aluminum-Joining-Plate-10-S-4151-N-/330343501054?pt=LH_DefaultDomain_0&hash=item4cea01ccfe	
3382 N	Ebay	$ 5.25	4	http://www.ebay.com/itm/8020-T-Slot-Hardware-1-4-20-Economy-T-Nut-10-Series-3382-25-Pcs-N-/330861775900?pt=LH_DefaultDomain_0&hash=item4d08e60c1c	
92196A537	McMaster Carr	$ 7.31	3	mcmaster.com	
