# Supplementary material for: Instrument for Real-Time Digital Nucleic Acid Amplification on Custom Microfluidic Devices
Source: PLoS One. 2016 Oct 19;11(10):e0163060. doi: 10.1371/journal.pone.0163060 (PMC5070811; doi:10.1371/journal.pone.0163060)
Supplement: S4 File — A derivation of the convective cooling rate used in the device temperature simulations. (PDF) [file pone.0163060.s004.pdf]

**Supporting Information S4.**

**Derivation of convective cooling rate.** A derivation of the convective cooling rate used in the device temperature simulations.

The heat transfer coefficient of the SlipChip modeled in Comsol was calculated based on eq. 1

$$h = {Nu * k}/{cl} \qquad \text{eq. 1}$$

where *h* is the average heat transfer coefficient, *Nu* is the Nusselt Number, *k* is the thermal conductivity of the fluid, and *cl* is the characteristic dimension. The characteristic dimension is calculated based on eq. 2

$$cl = {W * L}/{2W + 2L} \qquad \text{eq. 2}$$

where *W* is the width of the plate, and *L* is the length of the plate. This can also be written as the plate area divided by the perimeter. The Nusselt Number is calculated based on eq. 3

$$Nu = 0.54(Gr * Pr)^{0.25} \qquad \text{eq. 3}$$

where *Gr* is the Grashof Number and *Pr* is the Prandtl Number. The Prandtl Number is calculated based on eq. 4

$$\mathrm{Pr} = \nu * \alpha \qquad \text{eq. 4}$$

where *v* is the kinematic viscosity of the air, and *α* is the thermal diffusivity of the air. The thermal diffusivity is calculated based on eq. 5

$$\alpha = {k}/{\rho * c_p} \qquad \text{eq. 5}$$

where *k* is the thermal conductivity, ρ is the density, and $c_p$ is the specific heat capacity of the air. The Grashof number is calculated based on eq. 6

$$Gr = {g * B * (T_p - T_a) * cl^3}/{\nu^3} \qquad \text{eq. 6}$$

where *g* is gravity, *B* is the coefficient of thermal expansion, $T_p$ is the temperature of the chip, and $T_a$ is the temperature of the air. The density of the air must be adjusted as well for the film temperature of air above the chip which is calculated based on eq. 7 and eq. 8

$$T_f = {T_p + T_a}/{2} \qquad \text{eq. 7}$$

where $T_f$ is the temperature of the film and

$$\rho = \rho_{ref}\ {T}/{T_f} \qquad \text{eq. 8}$$

where $\rho_{ref}$ is the reference density, and the temperatures are in Kelvin.

The constants used to calculate the heat transfer coefficient are shown in Table 1.

**Table S1**

| | |
|---|---|
| $C_p$ | 1007 J/kg*C |
| B | 0.0034 1/K |
| k | 0.0261 W/m*C |
| ν | $1.83*10^{-5}$ $m^2$/sec |
| ρ | 1.201 kg/$m^3$ |
| W | 0.0762 m |
| L | 0.0254 m |
| $T_p$ | 98 C |
| $T_a$ | 25 C |

Using the constants in Table 1, and the equations above a heat transfer coefficient of 8.7 W/$m^2$C is calculated. As the temperature drops, the heat transfer coefficient goes down as well. Therefore, to account for the fact that there is likely some amount of air flow over the surface of the chip adding to the cooling rate, a set figure of 10 W/$m^2$C for the heat transfer was used in the COMSOL simulations.
