# Supplementary material for: Instrument for Real-Time Digital Nucleic Acid Amplification on Custom Microfluidic Devices
Source: PLoS One. 2016 Oct 19;11(10):e0163060. doi: 10.1371/journal.pone.0163060 (PMC5070811; doi:10.1371/journal.pone.0163060)
Supplement: S5 File — A brief guide detailing how to use the collection and analysis software. (PDF) [file pone.0163060.s005.pdf]

Supporting Information S5.

Software users' guide. A brief guide detailing how to use the collection and analysis software.

Operation of the collection software:

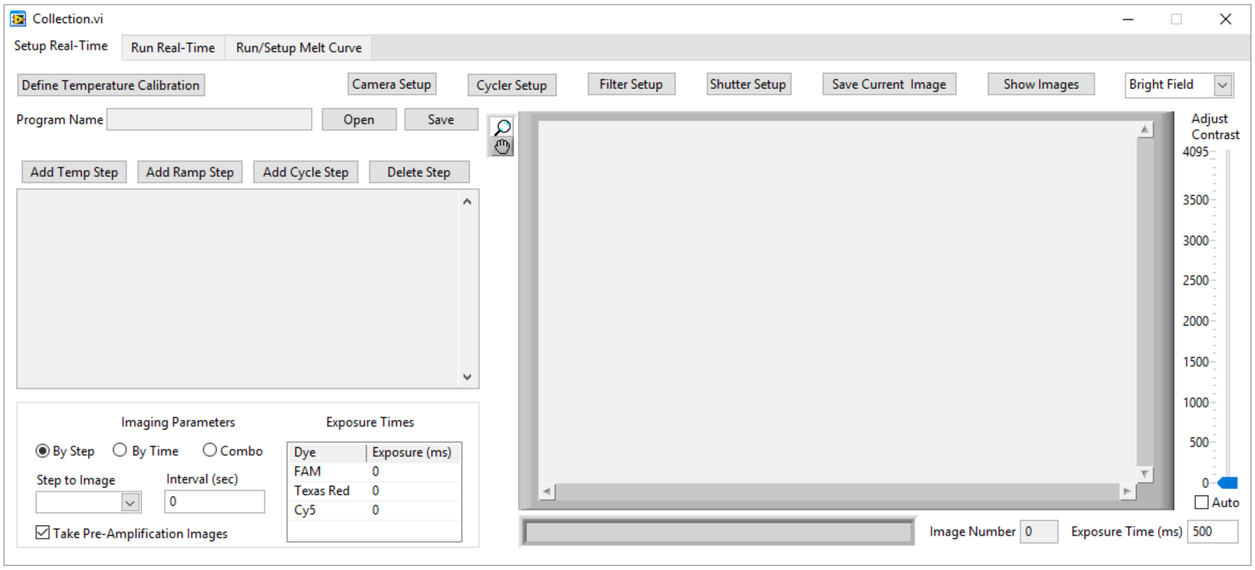

Real-Time experiments are set up using the page shown above.

1) Temperature parameters are input using the buttons on the top left. A temperature program can be saved using the buttons above the temperature input buttons.
2) Experimental parameters are input below the temperature program.
3) Each dye can have a different exposure time, and more than one exposure time can be used for each of the dyes, and multiple dyes can be used for imaging by selecting the ones desired.
4) The camera can be focused using the readout to the right of the page.
5) The buttons along the top can be used to setup and verify each individual piece of equipment.

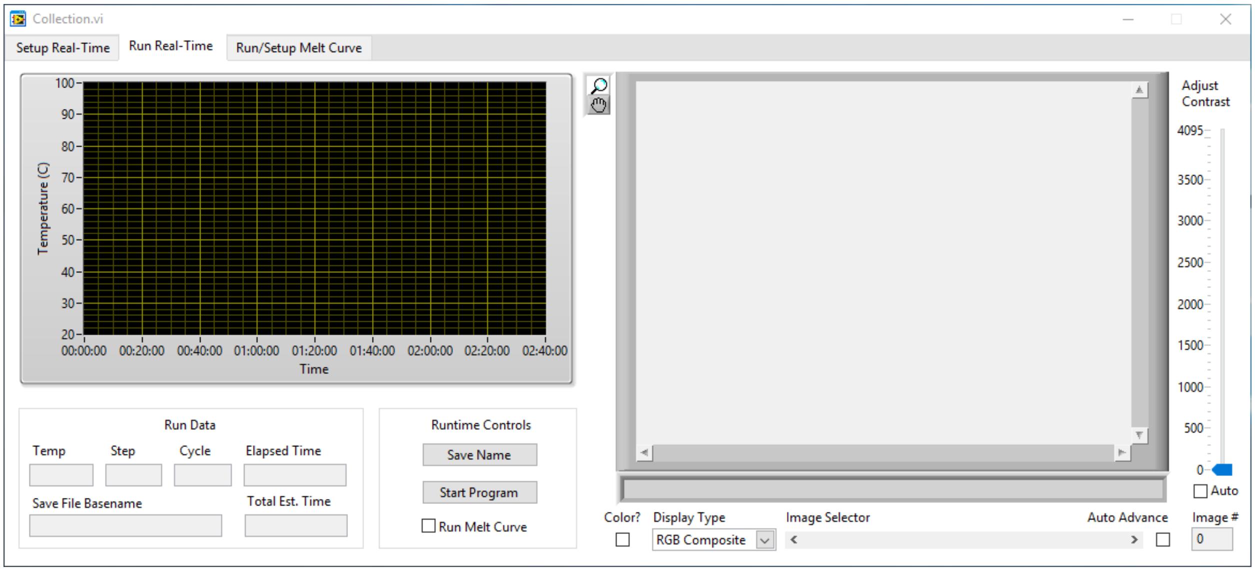

Real-time experiments are started using the page shown above

1) The name and location of the save is denoted using the button in the middle bottom of the page.

2) After all parameters are defined, the program can be started using the “start program” button.
3) If a melt-curve is desired, the box can be checked before starting the program.
4) While the program is running, the graph is updated with the temperature profile, and the “run data” parameters are updated in real-time.
5) Each image collected can be viewed using the viewer on the right.

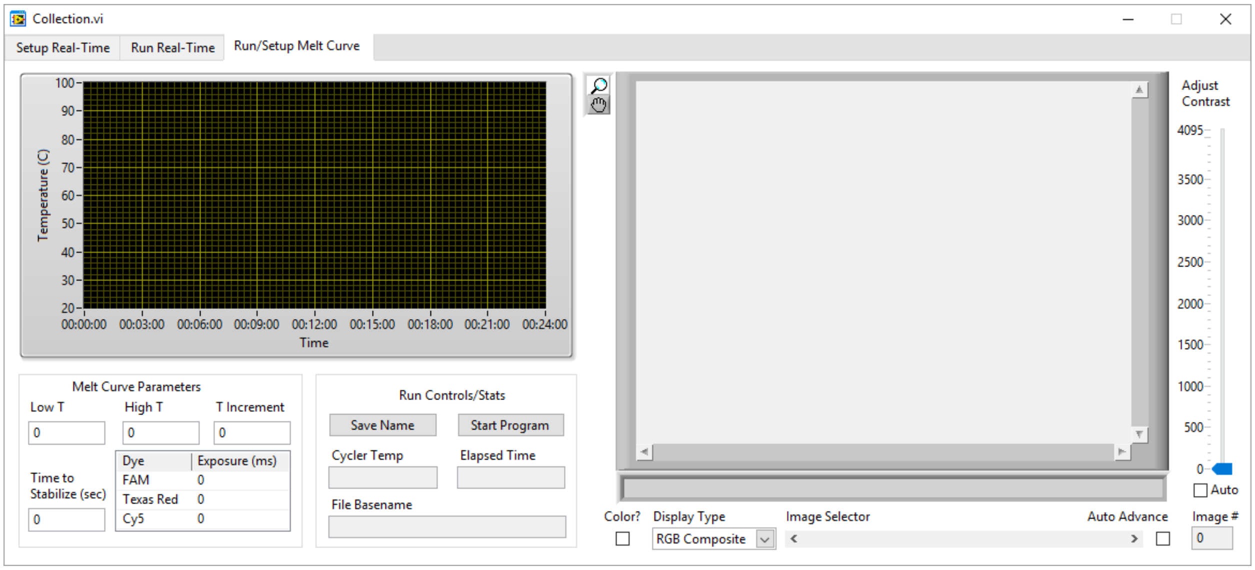

Melt curve experiments can be setup using the following page.

1) Melt curve experiments are defined with a low temperature, a high temperature, a temperature increment, a stabilization time, and exposure times for desired dyes. More than one dye can be used in the melt-cure generation.
2) The save name and location is defined using the “save name” button.
3) If a standalone melt-curve is desired, it can be started using the “start program” button. This is unnecessary if the melt curve is collected after the real-time curve with the box checked.’
4) Each melt curve image can be viewed using the viewer on the right.

Operation of the Analysis Software

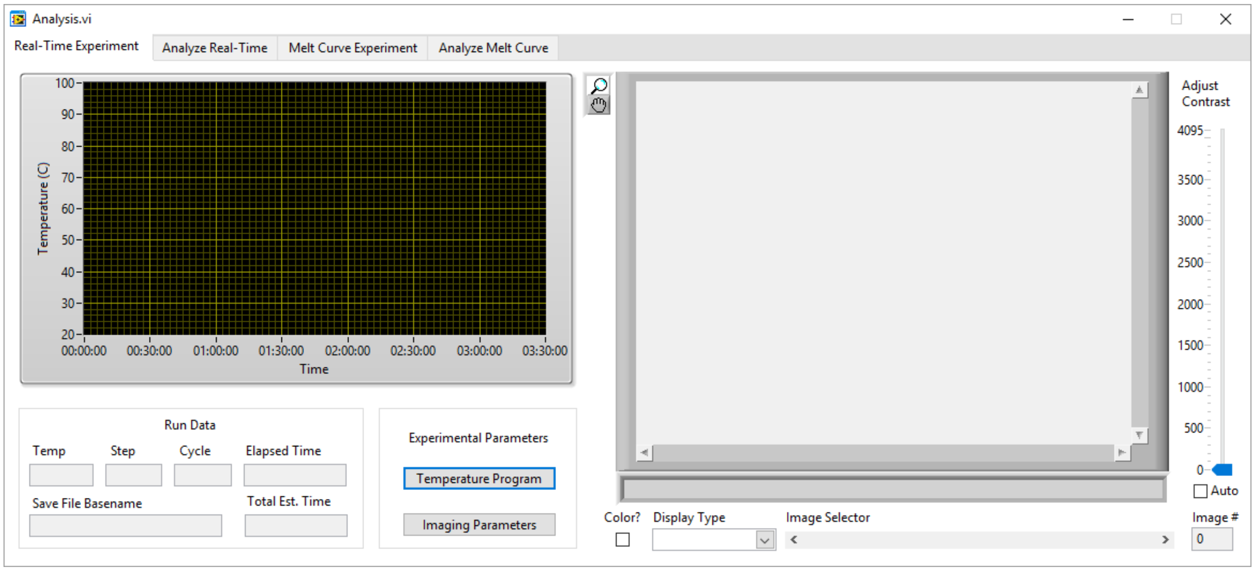

Real-time experiments can be viewed using this page.

1) After an experiment is loaded using the “analyze real-time” tab the parameters used, temperature profile, and raw images can be viewed on this page.

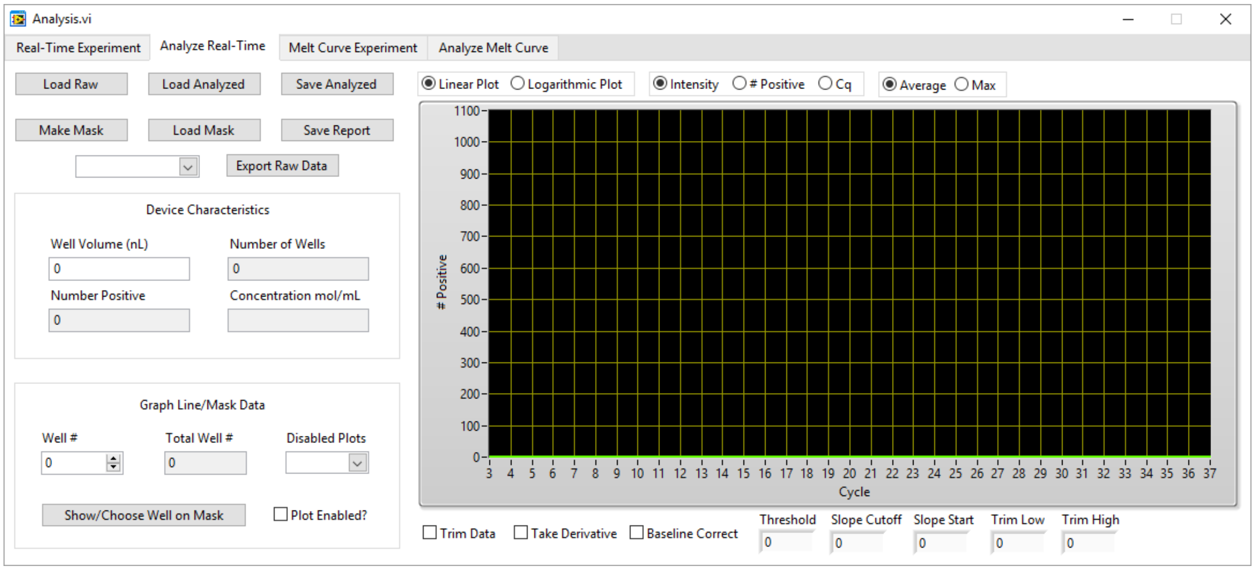

Real-time analyses are performed using this page

1) Raw experiments are loaded using the “load raw” button
2) Previously analyzed experiments are loaded using the “load analyzed” button
3) After analyzing raw data, the progress can be saved using the “save analyzed” button
4) A mask used for defining digital reaction locations can be made using the “make mask” button
5) A previous mask can be loaded using the “load mask” button
6) A report of the analysis can be generated using the “save report” button
7) Raw data in different channels can be exported using the “export raw data” button
8) Concentrations are automatically calculated after inputting the well volumes used in the “well volume” box and setting thresholds
9) Different real-time curves can be correlated to their location on the device as well as “hidden” using the section in the lower left
10) Parameters for baseline correcting, thresholding, and plotting the data are located above and below the plot

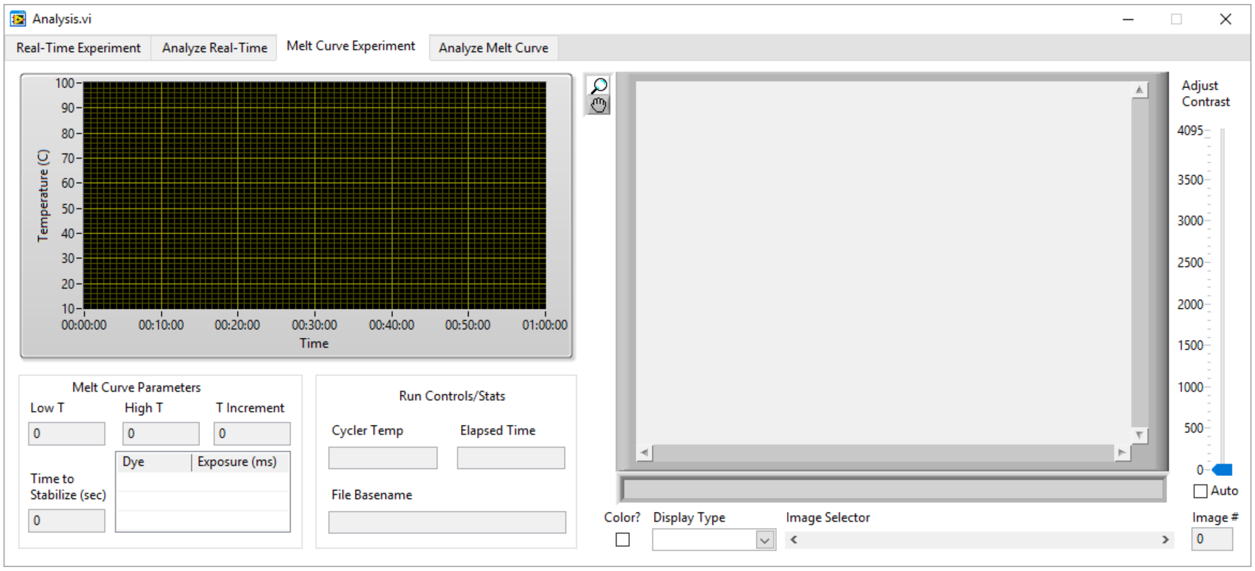

Akin to the real-time experiment tab, melt curve parameters are shown here after loading the raw data from the “analyze melt curve” tab

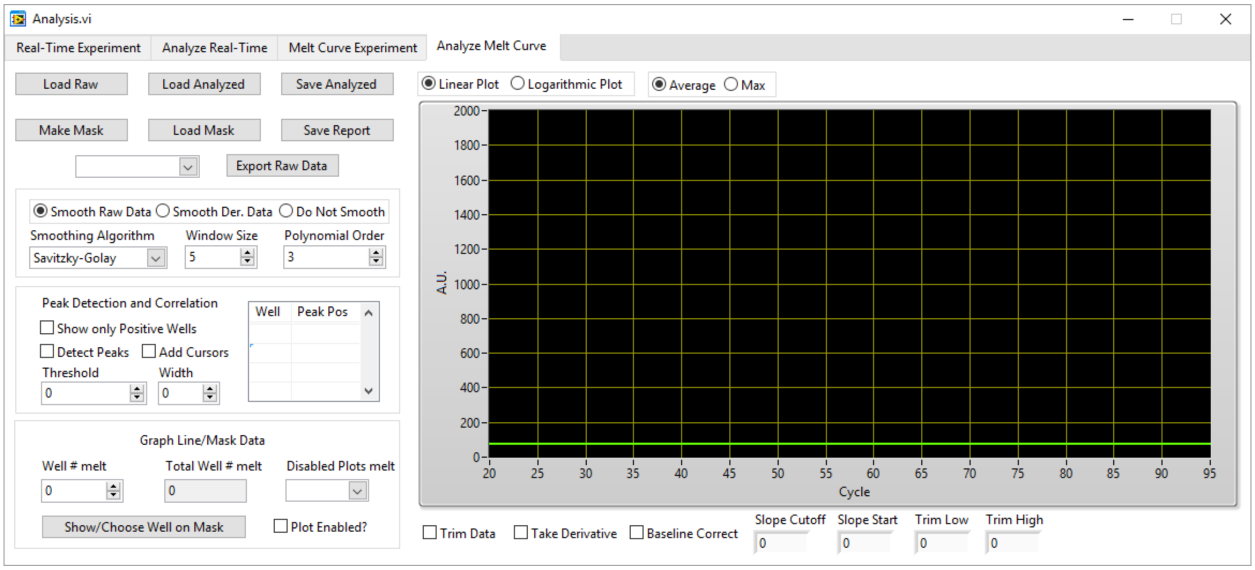

Melt curve experiments can be analyzed using this page

1) The buttons in the top left section of the page perform the same functions on the melt data as they performed on the real-time data. It is of note, that the same mask can be used for both datasets
2) Data smoothing can be performed using the parameters and algorithms defined in the section below the “export raw data” button
3) Peak detection and melt temperatures can be calclulated using the section below the data smoothing. It is of note, that if real-time anlaysis data is loaded into the program, it can be correlated with the melt temperature data using the “show only positive wells” button. This button only works if the same mask is used for both datasets
4) All other features are identical to the analyze real-time page.
